# Protocol for a pilot randomised, double-blind, placebo-controlled trial for assessing the feasibility and efficacy of faecal microbiota transplantation in adolescents with refractory irritable bowel syndrome: FAIS Trial

**DOI:** 10.1136/bmjpo-2020-000689

**Published:** 2020-08-20

**Authors:** Judith Zeevenhooven, Clara Marieke Andrea de Bruijn, Arine Vlieger, Max Nieuwdorp, Marc Alexander Benninga

**Affiliations:** 1 Paediatric Gastroenterology, Emma Childrens Hospital AMC, Amsterdam, North Holland, The Netherlands; 2 Amsterdam UMC, University of Amsterdam, Gastroenterology and Hepatology, Amsterdam Gastroenterology Endocrinology Metabolism Research Institute, Amsterdam, the Netherlands; 3 Amsterdam University Medical Centers, Location Academic Medical Center/Emma Children’s Hospital, Amsterdam Reproduction & Development Research Institute, Amsterdam, the Netherlands; 4 Department of Paediatrics, Sint Antonius Hospital, Nieuwegein, The Netherlands; 5 Department of Internal Medicine, Amsterdam UMC—Locatie AMC, Amsterdam, North Holland, Netherlands; 6 Paediatric Gastroenterology and Nutrition, Emma Kinderziekenhuis AMC, Amsterdam, North Holland, Netherlands

**Keywords:** gastroenterology

## Abstract

**Background:**

Irritable bowel syndrome (IBS) is a common chronic medical condition, in both children and adults. Despite the availability of effective (non)pharmacological treatments, symptoms persist in a significant amount of patients with IBS. Faecal microbiota transplantation (FMT) may be an effective alternative treatment in adolescents with refractory IBS through manipulation of the intestinal microbiota.

**Methods and analysis:**

This randomised, placebo-controlled single-centre pilot study will assess feasibility and efficacy of FMT in 30 adolescents (16–21 years) with refractory IBS. Patients will be randomly allocated (1:1) to receive two allogeneic (healthy donor) or two autologous (own) faecal infusions at baseline and after 6 weeks. Primary outcomes will assess feasibility, including patient and donor recruitment, adherence and incidence rates of adverse events. To evaluate clinical efficacy, secondary outcomes will include the proportion of patients with at least >50% reduction of their abdominal pain intensity and frequency 12 weeks after the first FMT, and after 6-month and 12-month follow-up. Other outcomes comprise changes in faecal gut microbiota composition, quality of life, depression and anxiety, school or work absenteeism and adequate relief, measured directly after FMTs and after 6 and 12 months of follow-up.

**Discussion:**

This randomised controlled trial will investigate the feasibility and effectiveness of repetitive FMTs in adolescents with refractory IBS.

**Ethics and dissemination:**

The study is approved by the Medical Research Ethics Committees AMC (MEC-AMC) in the Netherlands.

**Trial registration number:**

NCT03074227.

**What is already known on this topic?**

- It is suggested that irritable bowel syndrome (IBS) symptoms are generated through an effect of the microbiome on the intestinal barrier, enteroendocrine system, the immune system and the gut–brain axis.
- Faecal microbiota transplantation (FMT), administered via a nasoduodenal tube, is a new treatment regimen which modifies the gut microbiome through replacement of the patient microbiome by that of a healthy donor.

**What this study hopes to add?**

- This randomised controlled trial will investigate the feasibility and effectiveness of repetitive FMTs in adolescents with refractory IBS.
- This study will enable us to analyse in detail which microbiota components might predict a positive response to FMT.

## Background

Irritable bowel syndrome (IBS) according to the Rome IV criteria (box 1) is a common chronic medical condition, with worldwide pooled prevalence rates in adults and children ranging from 5.8% to 17.5% and 6.2% to 11.9%, respectively.^1 2^ Some studies report a peak prevalence in adolescents (12–19 years).^3^ IBS impairs daily life, as patients report a decreased quality of life,^4 5^ high work or school absence^6 7^ and a higher risk to develop depressive and anxiety disorders compared with healthy controls.^7 8^ Consequently, healthcare costs are substantial.^9 10^

Box 1 **Rome IV criteria: irritable bowel syndrome^41^**

Diagnostic criteria must include all of the following*

- Abdominal pain at least 4 days per month associated with one or more of the following:
  - Related to defecation
  - A change in frequency of stool
  - A change in form (appearance) of stool
- In children with constipation, the pain does not resolve with resolution of the constipation (children in whom the pain resolves have functional constipation, not irritable bowel syndrome).
- After appropriate evaluation, the symptoms cannot be fully explained by another medical condition.

*Criteria fulfilled for at least 2 months before diagnosis.

Standard medical care for IBS consists of education, reassurance and simple dietary and behavioural advices.^11 12^ Subsequently, either a pharmacological (tricyclic antidepressants, peppermint oil, linaclotide and lubiprostone) or non-pharmacological treatment (hypnotherapy and cognitive–behavioural therapy) can be considered.^11–14^ In the treatment of adolescent patients with IBS, evidence for the efficacy of pharmacological agents is scarce and inconclusive.^15^ In addition, some interventions that modify the microbiome, such as rifaximin or particular strains of probiotics, appear to have beneficial effects in adult patients with IBS,^16^ and in adolescent patients as well.^15 17^ Some low-quality evidence exists for the dietary low in fermentable oligosaccharides, disaccharides and monosaccharides and polyols (FODMAP) intervention in adult and adolescent patients with IBS.^17 18^ Finally, some psychological therapies, such as hypnotherapy, relaxation therapy and cognitive–behavioural therapy, are proven to be effective treatments for IBS.^13 19^ Despite these available treatments, symptoms may persist in some patients with IBS.^20^ These patients with IBS can be considered as therapy resistant (refractory) and might benefit from another potential treatment. Recent publications in children and adults indicate that altered gut microbiota may play an important role in the pathophysiology of IBS.^21–23^ Symptoms may be generated through effects of the microbiome on the intestinal barrier, enteroendocrine system, the immune system, the gut–brain axis, regulation of bile acid deconjugation, but also via diet derived metabolites produced by the microbiota.^24 25^ Therefore, manipulation of the intestinal microbiota by faecal microbiota transplantation (FMT), which modifies the gut microbiome through replacement of the patient microbiome by that of a healthy donor, in refractory patients with IBS can potentially have beneficial effects on IBS symptoms. FMT has been shown to be highly effective in treating adults with recurrent *Clostridium difficile* infection^26^ and yielded promising results in patients with ulcerative colitis^27^ and metabolic syndrome.^28^ For IBS, six randomised controlled trials (RCTs) on efficacy of FMT have been performed in adults.^29–31^ Two recent meta-analyses on these trials concluded that FMT versus placebo yielded no significant improvement in IBS symptoms, but results were hampered by significant inconsistency due to important differences in FMT methodology.^29 30^ To the best of our knowledge, no study has yet assessed the effect of FMT in adolescents with refractory IBS. Therefore, the objective of this RCT is to assess feasibility and effectiveness of FMT in adolescents with refractory IBS.

## Methods

### Trial design

**Figure 1:**
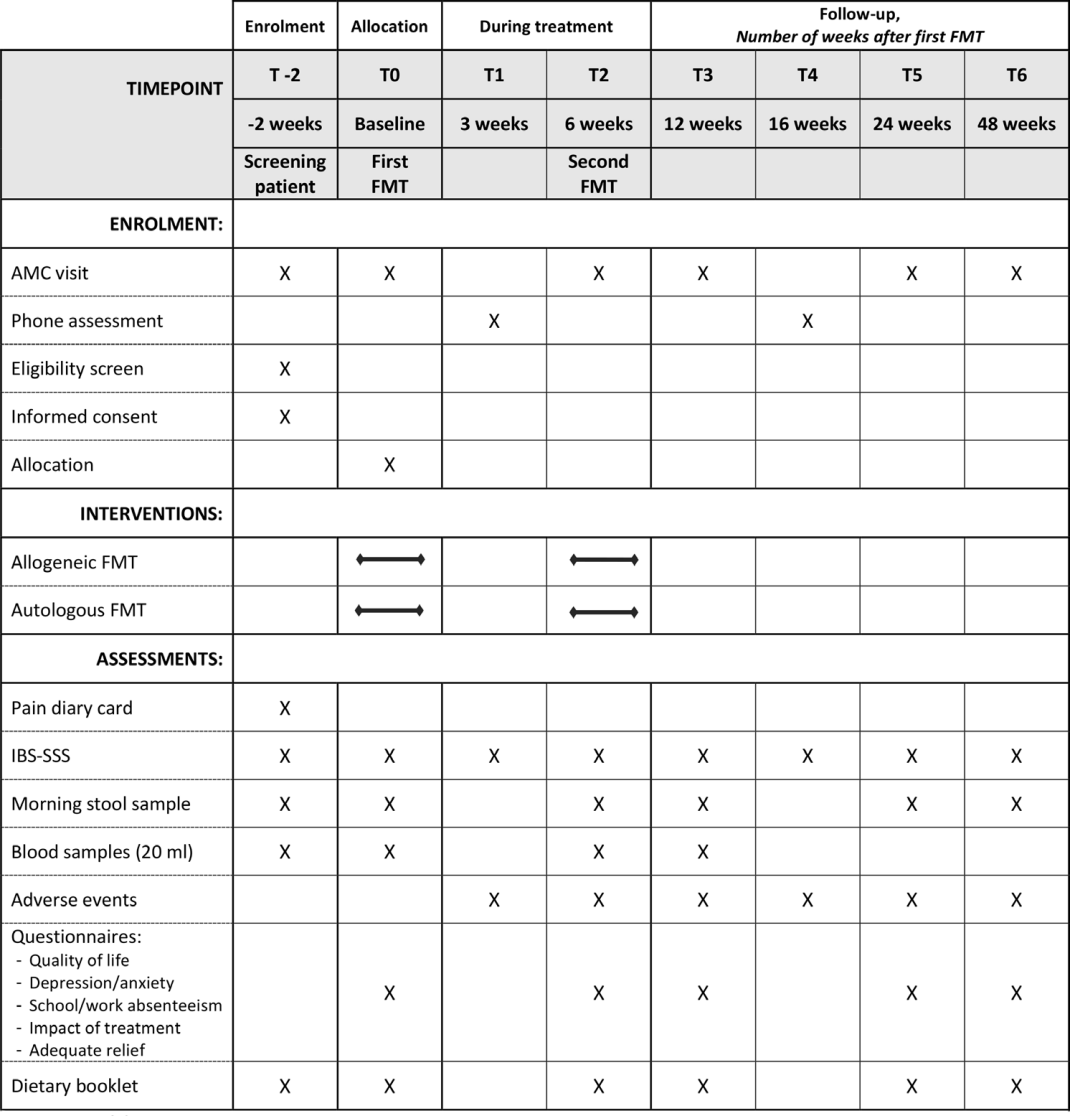
Trial design. After adolescents sign the informed consent form (T-2), patients complete the baseline pain diary, the Irritable Bowel Syndrome Severity Scoring System (IBS-SSS) and deliver stool samples and blood samples for eligibility screening. At T0, adolescents are randomised in the allogeneic or autologous faecal microbiota transplantation (FMT) group.

The Faecal Administration in refractory Irritable bowel Syndrome trial is a double-blind, randomised, placebo-controlled single-centre pilot study. We aim to enrol 30 adolescents aged between 16 and 21 years, with refractory IBS (box 2). After randomisation, patients will either receive two allogenic FMTs from a healthy donor or two autologous FMTs at baseline and after 6 weeks. The flow of the study protocol is presented in figure 1.

Box 2 **Refractory irritable bowel syndrome (IBS)**

- IBS according to the Rome IV criteria.
- Symptoms are present for ≥12 months.
- Patients received adequate explanation, reassurance and dietary advice for their symptoms.
- There is an absence of response to a minimum of six sessions of psychological treatment, like hypnotherapy or cognitive–behavioural therapy.
- There is an absence of response to an adequate dose of at least one pharmacological agent tried for a minimum of 6 weeks.

#### Patient and public involvement

There was no involvement of patients or the public in the design of this RCT.

### Procedure

#### Recruitment

##### Patients

Patients from the outpatient clinic of the Amsterdam University Medical Centre (AUMC) will be recruited by their treating gastroenterologist. Furthermore, patients from other hospitals can be referred to the AUMC for participation in this study. In addition, patients will be recruited throughout the Netherlands with help of online advertisement through IBS patient associations. Patient enrolment began in September 2018.

##### Donors

Healthy faecal donors will be recruited through advertisement in the form of posters, intranet network and emails, and via word by mouth.

#### Participant screening

##### Patients

Eligible patients will be invited for a screening visit. Informed consent from the participants will be obtained by the clinical research coordinator. During the screening visit, adolescents will undergo routine laboratory testing to exclude underlying organic disorders (table 1). Furthermore, patients will fill out a pain diary.

**Table 1:** Specification of patient screening

**Faeces screening**	
Calprotectine	
**Bacteria**	
*Clostridium difficile*	
*Helicobacter pylori*	
**Parasites**	
*Giardia lamblia*	*Dientamoeba fragilis*
*Cryptosporidium* sp	*Blastocystis hominis*
*Entamoeba histolytica*	
**Other**	
Parasitic worm eggs	Protozoan cysts and oocysts
Larvae	
**Serum screening**	
**Haematology**	
Complete blood count	Alkaline phosphatase
C-reactive protein	Kreatinine
Bilirubine	Ureum
Aspartate aminotransferase	Estimated glomerular filtration rate
Alanine aminotransferase	Antitransglutaminase antibodies
Gamma-glutamyl transferase	IgA
Viruses*	
Cytomegalovirus	
Epstein-Barr virus	

*In case of seronegativity, a matching seronegative donor will be used for faecal microbiota transplantation.

##### Donors

Potential donors will be thoroughly screened according to the screenings protocol of the Netherlands Donor Faeces Bank.^32^ Potential donors have to complete an extensive questionnaire regarding risk factors for infectious diseases and factors potentially perturbing the intestinal microbiota. Exclusion criteria for donors are outlined under ‘Eligibility criteria’. If donors are considered eligible after completing the questionnaire, they will undergo serum and faeces laboratory testing to exclude potentially transmittable diseases (table 2).

**Table 2:** Specification of donor screening

**Faeces screening**	
Calprotectine	
**Bacteria**	
*Clostridium difficile*	*Yersinia enterocolitica*
*Helicobacter pylori*	*Plesiomonas shigelloides*
*Salmonella* sp	Pathogenic *Campylobacter* sp
*Shigella* sp	Shiga toxin-producing *Escherichia coli*
**Antibiotic-resistant bacteria**	
Vancomycin-resistant *Enterococcus*	Multidrug-resistant Gram-negative (MRGN) 3
Carbapenem-resistant *Enterobacteriaceae*	MRGN 4
Methicillin-resistant *Staphylococcus aureus*	Extended spectrum beta-lactamase-producing *Enterobactereacceae*
**Viruses**	
Hepatitis E	Rotavirus
Norovirus type I and II	Enterovirus
Astrovirus	Adenovirus non-41/41
Sapovirus	Parechovirus
Adenovirus type 40/41	COVID-19
**Parasites**	
*Giardia lamblia*	*Microsporidium* sp
*Cryptosporidium* sp	Blastocystis hominis*
*Entamoeba histolytica*	*Isospora* sp
*Dientamoeba fragilis*	*Cyclospora*
**Non-pathogenic parasites*†***	
*Entamoeba gingivalis*	*Endolimax nana*
*Entamoeba hartmanni*	*Iodamoeba bütschlii*
*Entamoeba coli*	*Entamoeba dispar*
*Entamoeba polecki*	*Entamoeba moshkovskii*
**Other**	
Parasitic worm eggs	Protozoan cysts and oocysts
Larvae	
**Serum screening**	
Haematology	
Complete blood count	Gamma-glutamyl transferase
Bilirubine	Alkaline phosphatase
C-reactive protein	Kreatinine
Aspartate aminotransferase	Ureum
Alanine aminotransferase	Estimated glomerular filtration rate
**Bacteria**	
Lues	
**Viruses**	
Hepatitis A	*Cytomegalovirus*
Hepatitis B	Epstein-Barr virus
Hepatitis C	Human T-lymphotropic virus
HIV	
**Parasites**	
*Strongyloides*	

*Exclusion of donor only if microscopically ‘much’ or ‘very much’ blastocystis are seen.

†Presence of only one non-pathogenic parasite is acceptable.

### Eligibility criteria

#### Patients

##### Inclusion criteria

- Age 16–21 years.
- Non-smokers.
- Ability to give informed consent.
- IBS diagnosis (box 1).
- Refractory symptoms (box 2).
- Average daily pain rate ≥30 mm on the pain component scale of the Irritable Bowel Syndrome Severity Scoring System (IBS-SSS).^33^

##### Exclusion criteria

Exclusion criteria are presented in online supplementary table 1.

10.1136/bmjpo-2020-000689.supp1 Supplementary data

#### Donors

##### Inclusion criteria

- Age≥16 years.
- Non-smokers.
- Ability to give informed consent.
- Body mass index 18–25 kg/m^2^.
- Regular morning stool pattern.

##### Exclusion criteria

Exclusion criteria are presented in online supplementary table 1.

### Randomisation, blinding and treatment allocation

Randomisation will be done by a computerised random-number generator in the Electronic Data Capture system Castor EDC in a 1:1 ratio to one of the following two treatment arms:

1. Allogeneic faecal infusions at t=0 weeks and t=6 weeks.
2. Autologous faecal infusions at t=0 weeks and t=6 weeks.

Randomly permuted blocks of size 2 and 4 will be used with no stratification. On the day of faecal transplantation, both patient and donors will deliver faeces produced that morning. Randomisation will be performed by one of the ‘randomisation assistants’, who is designated to this task. To guarantee blinding, the randomisation assistant will make sure the randomised treatment is not traceable to the donor or the patient. The blinded faeces will be brought to the laboratory, where the preparation of the faeces will be done by one of the investigators. Detailed information about the preparation process is outlined under ‘FMT procedure’. During the second FMT at 6 weeks, faeces will be processed according to the randomisation performed on the first transplantation day. The randomisation assistant is the only person who will know which treatment the patient will be given and will have no role in further parts of the study. The randomisation list will be kept under secured access by Castor EDC. In case of an emergency, the study treatment can be unblinded after consultation of the principal investigator.

### Intervention

#### FMT procedure

At baseline and at 6 weeks, patient and donor will collect a fresh morning stool sample in a small container and bring this to the AUMC for processing. On arrival of the patient in the hospital, a nasoduodenal tube will be positioned under direct imaging, with the Cortrak electromagnetic sensing device.^34^ After placement of the nasoduodenal tube, bowel lavage with 1.5–3.5 L of macrogol electrolytes (Klean-Prep) solution will be performed according to standard protocols to ensure complete bowel lavage. The amount of solution that is given depends on the rapidity by which the bowel is cleaned. Finally, a faecal suspension of 200 ml will be infused in the duodenum of the patient through the nasoduodenal tube.

#### Preparation of faecal infusion product

On the day of infusion, a fresh faeces sample (100–200 g on average) of either the donor (allogeneic) or patient (autologous) will be used. In case a patient is not able to provide a fresh morning faeces sample, the first faecal production after the start of bowel lavage with Klean-Prep is used as suitable faecal sample for further processing. Time of collection will be recorded. The faeces will be weighted and mixed with 200–400 mL saline (0.9% NaCl) until fully homogenised. Next, the faeces solution is poured through a double gauze and debris of large size will be removed. This step will be repeated. Afterwards, the homogenised solution will be decanted through a metal funnel into a 200 ml sterile plastic bottle. All steps are performed under a fume hood by one of the coinvestigators. Within 6 hours after production by the donor, the faeces will be installed through the nasoduodenal tube in the patient.

### Outcomes

All below-mentioned outcome measures apply to patients.

#### Primary outcome

The primary objective of this RCT is to assess the feasibility of our study protocol. This will be assessed by evaluating the process of patient recruitment and screening, the patient drop-out rate and the incidence rates of adverse events (AEs). Table 3 delineates the feasibility outcome measures and measurement instruments.

**Table 3:** Trial outcome measures and instruments

	Outcome measures	Instrument
Feasibility outcomes	Patient recruitment	Patient recruitment per month *patient/month recruited*
	Patient screening	Patient eligibility *% of patients*
	Patient drop-out	Patient drop-out rate after randomisation *% of patients*, including patient acceptance to accomplish repetitive faecal microbiota transplantations (FMTs)
	Serious adverse events related to FMT	Hospitalisation or increase of >100 points on pain component of Irritable Bowel Syndrome Severity Scoring System (IBS-SSS) *% of patients*
	Stool sample collection	Patients provide all necessary stool samples *% of the provided samples*, morning stool samples will be collected during all study visits
Efficacy outcomes	>50% reduction of abdominal pain intensity and pain frequency compared with baseline at 12 (T3), 24 (T5) and 48 (T6) weeks after first FMT	Pain component of IBS-SSS score^33^ With two questions, the severity and frequency of the abdominal pain on the last 10 days are measured. The IBS-SSS is the only symptom severity scale that has been responsive to treatment effects.^42^ It has been recommended as a good instrument to obtain information on specific IBS-related symptoms.^43^
	Change in gut microbiota composition	MiSeq Illumina Sequencing Morning stool samples will be collected to profile the faecal microbiota composition by sequencing of the V4 region of the 16S ribosomal RNA gene
	Change in gut mycobiome composition	Internal transcribed spacer (ITS) sequencing Morning stool sample will be collected to profile the faecal mycobiome composition by high-throughput rDNA sequencing of fungal ITS−1 regions
	Change in gut metabolome composition	Capillary electrophoresis time-of-flight mass spectrometry (CE-TOF-MS) Morning stool sample will be collected to profile the faecal metabolome composition by CE-TOF-MS
	Number of adverse events	Patient CRF
	Number of rescue medication	Patient CRF
	Total IBS-SSS score	IBS-SSS score^33^ The IBS-SSS is the only symptom severity scale that has been responsive to treatment effects.^42^ It has been recommended as a good instrument to obtain information on specific IBS-related symptoms.^43^
	Health-related quality of life	Irritable Bowel Syndrome—Quality of Life questionnaire^44^ This questionnaire is a 34-item assessment of the degree to which the IBS interferes with patient quality of life and consists of eight domains: dysphoria, interference with activities, body image, health worry, food avoidance, social reactions, sexual health and effect on relationships.^44^
	Generic quality of life	Medical Outcomes Study 36-item Short Form Health Survey (SF-36) The SF-36 questionnaire consists of 36 questions regarding eight dimensions of health perception: limitations in physical functioning, role limitation due to physical health problems, bodily pain, general health perception, vitality, social functioning, role limitations due to emotional limitations and mental health. A score between 0 (worst possible quality of life) and 100 (best possible quality of life) can be obtained. The reliability has been proven extensively for diverse patient groups and it is validated for the Dutch population.^45^ The SF-36 is described as adequate for persons 14 years of age and older.^46^
	Depression and anxiety	Hospital Anxiety and Depression Scale (HADS) The HADS is divided into two 7-item scales, with answers on a 4-point scale (0–3). Higher scores indicate a higher level of anxiety or depression (range 0–21). A scale score of ≥8 (cut-off score) indicates clinically significant anxiety or depression. The Dutch version of the HADS showed satisfactory validity and reliability.^47^
	Absence of school or work, healthcare resources and costs	Adapted version of the Dutch Health and Labor Questionnaire^48^ School or work absenteeism and indirect healthcare utilisation costs are measures by three items. Adolescents indicate whether they have been absent from school or work due to abdominal pain problems, and if yes, the amount of hours per week. For the indirect costs of healthcare utilisation, adolescents indicate additional costs they had due to symptoms of abdominal pain over the past 4 weeks.
	Impact of treatment	Adapted version of the Patient Satisfaction and Preference Questionnaire^49^ Impact of FMT treatment will be assessed using five questions, which are based on the Patient Satisfaction and Preference Questionnaire used in another RCT on FMT in patients with recurrent *Clostridium Difficile* infection.^49^The questions address thoughts on how unpleasant and how dirty participants find the idea of getting a faecal transplant.
	Adequate relief	One question: ‘’Did you have adequate relief of IBS symptoms (abdominal discomfort/pain, bowel habits, and other symptoms like nausea and bloating) over the past week?’’ (Yes/No)
	Plasma biomarkers Intestinal fatty acid-binding protein (I-FABP) Smooth muscle protein of 22 kDa (SM-22) Citrulline	Vena puncture EDTA vacuum tubes were used. All blood samples were centrifuged at 4000 revolutions/min, at 41°C for 15 min to obtain plasma. Plasma was immediately stored in aliquots at −80°C until analysis
	Safety parameters C-reactive protein Liver function Renal function	Vena puncture EDTA vacuum tubes were used. All blood samples were centrifuged at 4000 revolutions/min, at 41°C for 15 min to obtain plasma. Plasma was immediately stored in aliquots at −80°C until analysis
	Dietary intake	Dietary diary Dietary intake lists are filled out 7 days prior to each faecal sample collection.

CRF, case report form; RCT, randomised controlled trial.;

#### Secondary outcomes

Secondary objectives include the proportion of patients with >50% reduction of their abdominal pain intensity and pain frequency compared with baseline at t=12 weeks after the first FMT. This will be assessed with the pain component of the IBS-SSS.^33^ Table 3 also describes all secondary outcome measures.

### Participant timeline

Figure 1 displays the time schedule of enrolment, interventions, assessments and visits for participating patients.

### Sample size calculation

Since this is a pilot study, a reliable sample size calculation is not feasible. In accordance with recruitment recommendations,^35 36^ a minimum of 15 patients per treatment group will be included. In addition, based on accumulated evidence with 16S rRNA sequencing using MiSeq, Illumina Platform, a sample size of 20 individuals is normally enough to detect relevant differences in the microbiota. Hence, a total sample size of n=30 seems adequate. In order to reduce heterogeneity in faecal transplants, 1 donor will donate faeces to approximately 3 patients, which implicates that 5 donors are needed for 30 patients.

### Statistical analysis

All data will be analysed according to the intention-to-treat principle. Feasibility outcome measures will be presented as proportions at each time point throughout the trial. To assess the efficacy outcomes group differences will be calculated by a mean difference with a 95% CI, using an independent t-test for continuous variables with a parametric distribution or Mann-Whitney U test for continuous variables with a non-parametric distribution. Group differences for categorical variables will be calculated using Fisher’s exact statistics. In addition, data of continues variables will be analysed using mixed models to account for correlations of measurements within the same individual on several time points. Due to the small sample size, baseline values will not be incorporated in these analyses. Significance is set at α=0.05 in all analyses.

Microbiota composition of the faecal samples will be measured by 16S rRNA sequencing and specific genera/species are screened by qPCR. Alfa and beta diversity of faecal samples will be calculated. Cluster analysis and similarity of the microbiota profiles, expressed as Pearson correlation, will be assessed and compared between patients with IBS and healthy donors, between treatment groups and between responders and non-responders. In addition, short-chain fatty acids composition of the faecal samples will be measured.

### Monitoring

#### Data monitoring

In order to optimise safety of the study during inclusion, patient data will be disclosed to a data safety monitoring board (DSMB) when 50% of the intended sample size is attained and has reached 12 weeks follow-up. The advice(s) of the DSMB will be notified on receipt by the sponsor to the METC (Medical Ethics Research Committee of the AUMC in Amsterdam, the Netherlands) that approved the protocol. With this notification, a statement will be included indicating whether the advice will be followed.

#### Harms

The risks associated with participation in this RCT can be considered moderate, because of the minimal invasive treatment. Nasoduodenal tube positioning through a Cortrak electromagnetic sensing device carries a little risk of complications like aspiration, perforation or malpositioning. If there is any doubt of malposition of the tube, a plain abdominal X-ray will be performed. To prevent complications, patients with swallowing disorders will not be included in this study.

Recent meta-analyses on clinical outcomes of FMT in general concluded that no serious AEs were attributable to FMT.^29 37^ AEs were infrequent and mostly self-limiting (ie, diarrhoea, abdominal distension, nausea and vomiting) and no differences existed in the number of AEs between donor FMT and control patients.^29 37^ In our study, AEs will be monitored throughout the whole study. In order to make the risk for transmission of infectious diseases as small as possible, rescreening of the faecal donors will be performed according to table 4. In accordance to the legal requirements in the Netherlands (article 10, subsection 1, WMO), the investigator will inform the subjects and the reviewing accredited METC if harmful events occur. When there are indications that the disadvantage of participation may be significantly greater than was described in the research proposal, the study will be suspended pending a further positive decision by the accredited METC. The investigator will take care that all subjects are kept informed.

**Table 4:** Time interval of donor rescreening*

		Rescreening interval			
		Pre-FMT	4 weeks	8 weeks	26 weeks
Short rescreening questionnaire		x			
Extensive rescreening questionnaire					x
**Faeces screening**					
Calprotectine					x
Bacteria					x
Antibiotic-resistant bacteria	COVID-19			x	
Viruses					x
Parasites					x
Non-pathogenic parasites†					x
Other					x
**Serum screening**					
Haematology					x
Bacteria					x
Viruses					x
*Cytomegalovirus* (CMV)	Epstein-Barr virus (EBV)		x*		
Parasites					x

*For specification of screening items, see table 2: specification of donor screening.

†When a donor is seronegative for EBV IgG and/or CMV IgG.

FMT, faecal microbiota transplantation.

### Commencement of the trial

On 23 November 2017, the first study participant (in particular donor) was included in the trial. Until today, 58 potential donors were recruited of which 39 were included and started the screening procedure. Finally, a total of five donors were eligible to donate faeces. The first patient signed informed consent in August 2018. At time of writing, 15 adolescents were recruited and a total of 19 faecal transplantations have been performed.

## Discussion and conclusion

IBS is a chronic and disabling condition, which can pose great impact on daily life of patients, reflected in decreased quality of life,^4 5^ high work or school absence,^6 7^ a higher risk to develop depressive and anxiety disorders^7 8^ and substantial healthcare costs.^9 10^ Effective management strategies for adolescents and adults in the form of antidepressants, peppermint oil, cognitive–behavioural therapy, hypnotherapy, probiotics and low FODMAP diet exist. However, a subgroup of patients with IBS remains symptomatic. New effective treatment options for this subgroup are warranted and might be targeted on the altered microbiome in patients with IBS.^23^

Up to now, six RCTs have been performed to assess the effect of FMT in IBS in adults. Two trials assessed the effect of FMT administered by capsules, two evaluated the effect of FMT delivered by colonoscopy, one via gastroscope and one by nasojejunal tube.^30^ It appears that the efficacy of FMT is associated with the methodology of FMT and placebo, as donor faeces administered by colonoscopy, gastroscopy or nasojejunal tube demonstrated a clinically significant improvement in global IBS symptoms in comparison with autologous FMT via the same route, whereas stool capsules did not demonstrate any beneficial effect compared with placebo capsules.^30^

The present pilot study assesses the feasibility of FMT in adolescents with refractory IBS according to the Rome IV criteria. Furthermore, the efficacy of FMT on abdominal pain symptoms in these patients is explored. By designing this specific treatment protocol, a unique opportunity is created to investigate potential beneficial effects of restoring the gut microbiota composition on abdominal pain problems. Data of this study will help determine optimal study conditions and inform the choice of endpoints for future, larger size, double-blind RCTs on FMT in adolescents with IBS. Furthermore, this study will define preliminary efficacy results of the use of FMT in these patients. In addition, this study will enable us to analyse in detail which microbiota components might predict a positive response to FMT.

Our study has several strengths. First, the FMT will be administered via a nasoduodenal tube and it will be performed twice, since it has been demonstrated that this might enhance the effect of the FMT.^38^ Another strength is the 1-year follow-up, which allows us to assess the long-term effect of FMT.

A limitation of our study is the small sample size, which allows us to only encounter major effects of the FMT treatment. Furthermore, we decided to include patients with IBS regardless of subtype, leading to a heterogeneous patient population which may affect the efficacy results. Moreover, it is unclear what the effect of bowel lavage is on the efficacy of FMT and on microbiome composition. Studies with and without bowel preparations before FMT demonstrate great efficacy.^38 39^ In addition, it has been shown that bowel preparation can disrupt the colonic ecosystem where the overall microbiome composition recovers to baseline within 14 days after bowel cleansing.^40^ Our efficacy outcome measure is assessed at 12 weeks after the first FMT (and 6 weeks after the second FMT), which minimises the effect that the bowel cleansing can have on the microbiome composition.

In conclusion, the results of this trial will provide preliminary evidence for the use of FMT in adolescents with refractory IBS. The results will inform future larger, double-blind, placebo-controlled trials on the right sample size, on the feasibility of this study design, on efficacy outcome measures and on the potential of the microbiome to be a therapeutic target in IBS.

## Supplementary Material

Author's manuscript
