# Supplementary material for: Protocol for a pilot randomised, double-blind, placebo-controlled trial for assessing the feasibility and efficacy of faecal microbiota transplantation in adolescents with refractory irritable bowel syndrome: FAIS Trial
Source: BMJ Paediatr Open. 2020 Aug 20;4(1):e000689. doi: 10.1136/bmjpo-2020-000689 (PMC7443263; doi:10.1136/bmjpo-2020-000689)
Supplement: Supplementary data [file bmjpo-2020-000689supp001.pdf]

Supplementary Table 1. Exclusion criteria patients and donors

**Patients**
Use of systemic antibiotics in preceding 6 weeks
Use of probiotic treatment in preceding 6 weeks
Use of concomitant medication, including proton pomp inhibitors (PPI) and vasopressine medication. Pain medication in the form of Paracetamol or NSAIDs is allowed.
Current use of drugs which influence gastrointestinal motility (erythromycin, azithromycin, butyl scopolamine, domperidone, peppermint oil capsules, iberogast)
Current treatment by another health care professional for abdominal symptoms
Current treatment by psychologist or shrink for known anxiety or depression disorder
Known swallowing disorder
Known diagnosis of inflammatory bowel disease (i.e. Crohn's disease or ulcerative colitis)
Known concomitant organic gastrointestinal disease
Known diagnosis of an autoimmune disease (e.g. hypo- or hyperthyroidism, celiac disease, rheumatoid arthritis)
Condition leading to profound immunosuppression (HIV, infectious diseases leading to immunosuppression, bone marrow malignancies)
Known diagnosis of cystic fibrosis
Known diagnosis of porphyria
Known pregnancy or current lactation
Use of systematic chemotherapy
Life expectancy < 12 months
Current Intensive Care Unit-stay
XTC, amphetamine or cocaine abuse
Known intra-abdominal fistula
Signs of ileus, diminished passage
Allergy to macrogol or substituents, e.g. peanuts, shellfish
History of surgery:<br>o *Hemicolectomy (defined as: surgery resulting in a resection of > 0.5 of the colon)*<br>o *Presence of a pouch due to surgery*<br>o *Presence of stoma*
Insufficient knowledge of the Dutch language
**Donors**
Abnormal bowel motions, abdominal complaints or symptoms indicative of irritable bowel syndrome
An extensive travel behaviour
Higher risk of colonization with multidrug- resistant organisms including:<br>o *Health care workers*<br>o *Persons who have recently been hospitalized or discharged from long term care facilities*<br>o *Persons who regularly attend outpatient medical or surgical clinics*<br>o *Persons who have recently engaged in medical tourism*
Unsafe sex practice (assessed with standardized questionnaire)
Use of any medication including PPI
Antibiotic treatment in the past 12 weeks
A positive history/clinical evidence for inflammatory bowel disease (i.e. Crohn's disease or ulcerative colitis)
A positive history/clinical evidence for other gastrointestinal diseases, including chronic diarrhoea or chronic constipation

Patients receiving immunosuppressive medications or a positive history/clinical evidence for autoimmune disease including:<br>o *Type 1 diabetes*<br>o *Hashimoto hypothyroidism*<br>o *Graves hyperthyroidism*<br>o *Rheumatoid arthritis*<br>o *Celiac disease*
History of or present known malignant disease and/or patients who are receiving systemic anti-neoplastic agents
Known psychiatric disease (i.e. depression, schizophrenia, autism, Asperger's syndrome)
Known chronic neurological/neurodegenerative disease (e.g. Parkinson's disease, multiple sclerosis)
Positive blood tests for the presence of: HIV, HTLV, lues, Strongyloides
Active hepatitis A, B-, C- or E-virus infection or known exposure within recent 12 months
Acute infection with cytomegalovirus (CMV) or Epstein-Barr virus (EBV)
Chronic pain syndromes (e.g. fibromyalgia)
Major relevant allergies (e.g. food allergy, multiple allergies)
Recent (gastrointestinal) infection within last 6 months
Tattoo or body piercing placement within last 6 months
Alcohol abuse (>3 units/day)
Known risk of Creutzfeldt Jacob's disease
History of current use of IV drugs
History of treatment with growth factors
