# Supplementary material for: Protocol for a pilot randomised, double-blind, placebo-controlled trial for assessing the feasibility and efficacy of faecal microbiota transplantation in adolescents with refractory irritable bowel syndrome: FAIS Trial
Source: BMJ Paediatr Open. 2020 Aug 20;4(1):e000689. doi: 10.1136/bmjpo-2020-000689 (PMC7443263; doi:10.1136/bmjpo-2020-000689)
Supplement: Author's manuscript [file bmjpo-2020-000689.draft_revisions.pdf]

BMJ Paediatrics Open

BMJ Paediatrics Open is committed to open peer review. As part of this commitment we make the peer review history of every article we publish publicly available.

When an article is published we post the peer reviewers' comments and the authors' responses online. We also post the versions of the paper that were used during peer review. These are the versions that the peer review comments apply to.

The versions of the paper that follow are the versions that were submitted during the peer review process. They are not the versions of record or the final published versions. They should not be cited or distributed as the published version of this manuscript.

BMJ Paediatrics Open is an open access journal and the full, final, typeset and author-corrected version of record of the manuscript is available on our site with no access controls, subscription charges or pay-per-view fees (http://bmjpaedsopen.bmj.com).

If you have any questions on BMJ Paediatrics Open's open peer review process please email info.bmjpo@bmj.com

BMJ Paediatrics Open

Protocol for a pilot randomised, double-blind, placebo-controlled trial for assessing the feasibility and efficacy of faecal microbiota transplantation in adolescents with refractory irritable bowel syndrome: FAIS Trial

Journal:	*BMJ Paediatrics Open*
Manuscript ID	bmjpo-2020-000689
Article Type:	Protocol
Date Submitted by the Author:	01-Apr-2020
Complete List of Authors:	Zeevenhooven, Judith; Emma Childrens Hospital AMC, Paediatric Gastroenterology<br>de Bruijn, Clara; Emma Childrens Hospital AMC, Paediatric Gastroenterology<br>Vlieger, Arine; Sint Antonius Hospital, Department of Paediatrics<br>Nieuwdorp, Max; Amsterdam UMC - Locatie AMC, Department of Internal Medicine<br>Benninga, Marc; Emma Kinderziekenhuis AMC, Paediatric Gastroenterology and Nutrition
Keywords:	Gastroenterology

SCHOLARONE™
Manuscripts

I, the Submitting Author has the right to grant and does grant on behalf of all authors of the Work (as defined in the below author licence), an exclusive licence and/or a non-exclusive licence for contributions from authors who are: i) UK Crown employees; ii) where BMJ has agreed a CC-BY licence shall apply, and/or iii) in accordance with the terms applicable for US Federal Government officers or employees acting as part of their official duties; on a worldwide, perpetual, irrevocable, royalty-free basis to BMJ Publishing Group Ltd ("BMJ") its licensees and where the relevant Journal is co-owned by BMJ to the co-owners of the Journal, to publish the Work in this journal and any other BMJ products and to exploit all rights, as set out in our licence.

The Submitting Author accepts and understands that any supply made under these terms is made by BMJ to the Submitting Author unless you are acting as an employee on behalf of your employer or a postgraduate student of an affiliated institution which is paying any applicable article publishing charge ("APC") for Open Access articles. Where the Submitting Author wishes to make the Work available on an Open Access basis (and intends to pay the relevant APC), the terms of reuse of such Open Access shall be governed by a Creative Commons licence – details of these licences and which Creative Commons licence will apply to this Work are set out in our licence referred to above.

Other than as permitted in any relevant BMJ Author's Self Archiving Policies, I confirm this Work has not been accepted for publication elsewhere, is not being considered for publication elsewhere and does not duplicate material already published. I confirm all authors consent to publication of this Work and authorise the granting of this licence.

Confidential: For Review Only

Protocol for a pilot randomised, double-blind, placebo-controlled trial for assessing the feasibility and efficacy of faecal microbiota transplantation in adolescents with refractory irritable bowel syndrome: FAIS Trial

Authors

Judith Zeevenhooven[1], Clara M.A. de Bruijn[1], Arine M. Vlieger[2], Max Nieuwdorp[3], Marc A. Benninga[1]

Affiliations

1. Emma Children's Hospital, Amsterdam UMC, University of Amsterdam, Paediatric Gastroenterology, Hepatology and Nutrition, Amsterdam, The Netherlands
2. Department of Paediatrics, St. Antonius Hospital, Nieuwegein, The Netherlands
3. Department of Internal Medicine, Amsterdam UMC, University of Amsterdam

Address correspondence

Judith Zeevenhooven, Emma Children's Hospital, Amsterdam UMC, University of Amsterdam, Paediatric Gastroenterology, Room C2-312, PO Box 22700, 1100 DD Amsterdam, The Netherlands. Telephone: +3120-5662906. Email: j.zeevenhooven@amsterdamumc.nl

Word Count

Main text: 2503

Tables: 6

Figures: 1

Number of references: 50

Confidential: For Review Only

ABSTRACT

Background: Irritable bowel syndrome (IBS) is a common chronic medical condition, in both children and adults. Despite the availability of effective (non)pharmacological treatments, symptoms persist in a significant amount of IBS patients. Faecal microbiota transplantation (FMT) may be an effective alternative treatment in adolescents with refractory IBS through manipulation of the intestinal microbiota.

Methods and analysis: This randomised, placebo-controlled single-centre pilot study will assess feasibility and efficacy of FMT in 30 adolescents (16-21 years) with refractory IBS. Patients will be randomly allocated (1:1) to receive two allogeneic (healthy donor) or two autologous (own) faecal infusions at baseline and after 6 weeks. Primary outcomes will assess feasibility, including patient and donor recruitment, adherence, and incidence rates of adverse events. To evaluate clinical efficacy, secondary outcomes will include the proportion of patients with at least > 50% reduction of their abdominal pain intensity and frequency 12 weeks after the first FMT, and after 6 and 12 months follow-up. Other outcomes comprise changes in faecal gut microbiota composition, quality of life, depression and anxiety, school or work absenteeism and adequate relief, measured directly after FMTs and after 6 and 12 months of follow up.

Discussion: This is the first RCT to investigate the feasibility and effectiveness of repetitive FMTs in adolescents with refractory IBS.

Ethics and dissemination: The study is approved by the Medical Research Ethics Committees AMC (MEC-AMC) in the Netherlands.

Trial registration details: Clinical trials registration number is NCT03074227.

Keywords: Faecal microbiota transplantation, FMT, irritable bowel syndrome, IBS, therapy resistant, adolescents

Confidential: For Review Only

What is known about the subject
➢ It is suggested that IBS symptoms are generated through an effect of the microbiome on the intestinal barrier, enteroendocrine system, the immune system and the gut-brain axis<br>➢ Faecal Microbiota Transplantation (FMT) administered via a nasoduodenal tube, is a new treatment regimen which modifies the gut microbiome through replacement of the patient microbiome by that of a healthy donor
**What this study hopes to add**
➢ This is the first RCT to investigate the feasibility and effectiveness of repetitive FMTs in adolescents with refractory IBS<br>➢ This study will enable us to analyse in detail which microbiota components might predict a positive response to FMT

Confidential: For Review Only

BACKGROUND

Irritable bowel syndrome (IBS) according to the Rome IV criteria (Table 1) is a common chronic medical condition, with worldwide pooled prevalence rates in adults and children ranging from 5.8-17.5% and 6.2-11.9%, respectively.[1,2] Some studies report a peak prevalence in adolescents (12-19 years).[3] IBS impairs daily life, as patients report a decreased quality of life,[4,5] high work or school absence,[6,7] and a higher risk to develop depressive and anxiety disorders compared to healthy controls.[7,8] Consequently, healthcare costs are substantial.[9,10]

**Table 1. Rome IV criteria: Irritable Bowel Syndrome**[11]
**Diagnostic Criteria must include all of the following***
1. Abdominal pain at least 4 days per month associated with one or more of the following:<br>a. Related to defecation<br>b. A change in frequency of stool<br>c. A change in form (appearance) of stool
2. In children with constipation, the pain does not resolve with resolution of the constipation (children in whom the pain resolves have functional constipation, not irritable bowel syndrome)
3. After appropriate evaluation, the symptoms cannot be fully explained by another medical condition
**Criteria fulfilled for at least 2 months before diagnosis.*

Standard medical care for IBS consists of education, reassurance and simple dietary and behavioural advices.[12,13] Subsequently, either a pharmacological (tricyclic antidepressants (TCA), peppermint oil, linaclotide and lubiprostone) or non-pharmacological treatment (hypnotherapy and cognitive behavioural therapy) can be considered.[12–15] In the treatment of adolescent IBS patients, evidence for the efficacy of pharmacological agents is scarce and inconclusive.[16] In addition, some interventions that modify the microbiome, such as rifaximin or particular strains of probiotics, appear to have beneficial effects in IBS adult patients,[17] and in adolescent patients as well.[16,18] Some low quality evidence exists for the dietary low in fermentable oligo-, di- and monosaccharides and polyols (FODMAP) intervention in adult and adolescent IBS patients.[19,18] Finally, some psychological therapies, such as hypnotherapy, relaxation therapy and cognitive behavioural therapy are proven to be effective treatments for IBS.[14,20] Despite these available treatments, symptoms

Confidential: For Review Only

may persist in some IBS patients.[21] These IBS patients can be considered as therapy resistant (refractory) and might benefit from another potential treatment. Recent publications in children and adults indicate that altered gut microbiota may play an important role in the pathophysiology of IBS.[22–24] Symptoms may be generated through effects of the microbiome on the intestinal barrier, enteroendocrine system, the immune system, the gut-brain axis, regulation of bile acid deconjugation, but also via diet derived metabolites produced by the microbiota.[25,26] Therefore, manipulation of the intestinal microbiota by faecal microbiota transplantation (FMT), which modifies the gut microbiome through replacement of the patient microbiome by that of a healthy donor, in refractory IBS patients can potentially have beneficial effects on IBS symptoms. FMT has been shown to be highly effective in treating adults with recurrent *Clostridium difficile* infection [27] and yielded promising results in patients with ulcerative colitis [28] and metabolic syndrome.[29] For IBS, six randomized controlled trials (RCTs) on efficacy of FMT have been performed in adults.[30–32] Two recent meta-analyses on these trials concluded that FMT versus placebo yielded no significant improvement in IBS symptoms, but results were hampered by significant inconsistency due to important differences in FMT methodology.[30,31] To the best of our knowledge, no study has yet assessed the effect of FMT in adolescents with refractory IBS. Therefore, the objective of this RCT is to assess feasibility and effectiveness of FMT in adolescents with refractory IBS.

Confidential: For Review Only

METHODS

Trial design

The Faecal Administration in refractory Irritable bowel Syndrome (FAIS) trial is a double-blind, randomised, placebo-controlled single-centre pilot study. We aim to enrol 30 adolescents aged between 16 and 21 years, with refractory irritable bowel syndrome (Table 2). After randomisation, patients will either receive two allogenic faecal microbiota transplantations (FMTs) from a healthy donor or two autologous FMTs at baseline and after 6 weeks. The flow of the study protocol is presented in Figure 1.

Table 2. Refractory Irritable Bowel Syndrome
1. Irritable bowel syndrome (IBS) according to the Rome IV criteria
2. Symptoms are present for ≥ 12 months
3. Patients received adequate explanation, reassurance and dietary advice for their symptoms
4. There is an absence of response to a minimum of six sessions of psychological treatment, like hypnotherapy or cognitive behavioural therapy
5. There is an absence of response to an adequate dose of at least one pharmacological agent tried for a minimum of six weeks

Patient and public involvement

There was no involvement of patients or the public in the design of this RCT.

Procedure

Recruitment

<u>Patients</u>

Patients from the outpatient clinic of the Amsterdam University Medical Centre (AUMC) will be recruited by their treating gastroenterologist. Furthermore, patients from other hospitals can be referred to the AUMC for participation in this study. In addition, patients will be recruited throughout

Confidential: For Review Only

the Netherlands with help of online advertisement through IBS patient associations. Patient enrolment began in September 2018.

Donors

Healthy faecal donors will be recruited through advertisement in the form of posters, intranet network and emails, and via word by mouth.

Participant screening

Patients

Eligible patients will be invited for a screening visit. Informed consent from the participants will be obtained by the clinical research coordinator. During the screening visit, adolescents will undergo routine laboratory testing to exclude underlying organic disorders (Table 3). Furthermore, patients will fill out a pain diary.

**Table 3. Specification of patient screening**	
**Faeces screening**	
Calprotectine	
Bacteria	
Clostridium difficile	
Helicobacter pylori	
Parasites	
Giardia lamblia	Dientamoeba fragilis
Cryptosporidium spp.	Blastocystis hominis
Entamoeba histolytica	
Other	
Parasitic worm eggs	Protozoan Cysts and Oocysts
Larvae	
**Serum screening**	
Hematology	
Complete Blood Count (CBC)	Alkaline phosphatase (AF)
C-reactive protein (CRP)	Kreatinine
Bilirubine	Ureum
Aspartate aminotransferase (ASAT)	Estimated Glomerular Filtration Rate (EGFR)
Alanine aminotransferase (ALAT)	Anti-transglutaminase antibodies
Gamma-glutamyl transferase (GGT)	IgA
Viruses*	

Confidential: For Review

Cytomegalovirus (CMV)
Epstein-Barr Virus (EBV)
* In case of seronegativity, a matching seronegative donor will be used for FMT<br><br>AF = Alkaline phosphatase; ALAT = Alanine aminotransferase; ASAT = Aspartate aminotransferase; CBC = complete blood count; CMV = cytomegalovirus; CRP = C-reactive protein; EGFR = Estimated Glomerular Filtration Rate; EBV = Epstein-Barr virus; GGT = Gamma-glutamyl transferase;

Donors

Potential donors will be thoroughly screened according to the screenings protocol of the Netherlands Donor Faeces Bank (NDFB).[33] Potential donors have to complete an extensive questionnaire regarding risk factors for infectious diseases and factors potentially perturbing the intestinal microbiota. Exclusion criteria for donors are outlined under ‘Eligibility criteria’. If donors are considered eligible after completing the questionnaire, they will undergo serum and faeces laboratory testing to exclude potentially transmittable diseases (Table 4).

Table 4. Specification of donor screening

**Faeces screening**	
Calprotectine	
Bacteria	
Clostridium difficile	Yersinia enterocolitica
Helicobacter pylori	Plesiomonas shigelloides
Salmonella spp	Pathogenic Campylobacter Spp.
Shigella spp.	Shiga toxin-producing Escherichia coli (STEC)
Antibiotic Resistant Bacteria	
Vancomycin-resistant Enterococcus (VRE)	Multidrug-resistant Gram-negative (MRGN) 3
Carbapenem-resistant Enterobacteriaceae (CRE)	MRGN 4
Methicillin-resistant Staphylococcus aureus (MRSA)	Extended spectrum beta-lactamase (ESBL)-producing Enterobactereacceae
Viruses	
Hepatitis E	Rotavirus
Norovirus Type I and II	Enterovirus
Astrovirus	Adenovirus non-41/41
Sapovirus	Parechovirus
Adenovirus type 40/41	
Parasites	
Giardia lamblia	Microsporidium spp.
Cryptosporidium spp.	Blastocystis hominis*
Entamoeba histolytica	Isospora spp.
Dientamoeba fragilis	Cyclospora
Non-pathogenic parasites*	
Entamoeba gingivalis	Endolimax nana

Confidential: For Review Only

Entamoeba hartmanni	Iodamoeba bütschlii
Entamoeba coli	Entamoeba dispar
Entamoeba polecki	Entamoeba moshkovskii
Other	
Parasitic worm eggs	Protozoan Cysts and Oocysts
Larvae	
**Serum screening**	
Hematology	
Complete Blood Count (CBC)	Gamma-glutamyl transferase (GGT)
Bilirubine	Alkaline phosphatase (AF)
C-reactive protein (CRP)	Kreatinine
Aspartate aminotransferase (ASAT)	Ureum
Alanine aminotransferase (ALAT)	Estimated Glomerular Filtration Rate (EGFR)
Bacteria	
Lues	
Viruses	
Hepatitis A	Cytomegalovirus (CMV)
Hepatitis B	Epstein-Barr Virus (EBV)
Hepatitis C	Human T-lymphotropic virus (HTLV)
Human immunodeficiency viruses (HIV)	
Parasites	
Strongyloides	

* Exclusion of donor only if microscopically "much" or "very much" blastocystis are seen
** Presence of only one non-pathogenic parasite is acceptable

CRE = Carbapenem-Resistant Enterobacteriaceae; ESBL = Extended spectrum beta-lactamase; MRGN = multidrug-resistant Gram-negative; MRSA = Methicillin-resistant Staphylococcus aureus; STEC = Shigatoxine-Producerende E. Coli; VRE = Vancomycin resistant enterococ; AF = Alkaline phosphatase; ALAT = Alanine aminotransferase; ASAT = Aspartate aminotransferase; CBC = complete blood count; CMV = cytomegalovirus; CRP = C-reactive protein; EGFR = Estimated Glomerular Filtration Rate; EBV = Epstein-Barr virus; GGT = Gamma-glutamyl transferase; HIV = Human immunodeficiency viruses; HTLV = Human T-lymphotropic virus

For peer review only

Eligibility criteria

<u>Patients</u>

Inclusion criteria

- Age 16-21 years
- Non-smokers
- Ability to give informed consent
- IBS diagnosis (Table 1)
- Refractory symptoms (Table 2)
- Average daily pain rate ≥30mm on the pain component scale of the IBS-SSS[34]

Exclusion criteria

Exclusion criteria are presented in Supplementary Table 1.

<u>Donors</u>

Inclusion criteria

- Age ≥16 years
- Non-smokers
- Ability to give informed consent
- BMI 18-25 kg/m2
- Regular stool pattern

Exclusion criteria

Exclusion criteria are presented in Supplementary Table 1.

Randomisation, blinding and treatment allocation

Confidential: For Review Only

Randomisation will be done by a computerised random-number generator in the Electronic Data Capture system Castor EDC in a 1:1 ratio to one of the following two treatment arms:

1. Allogeneic faecal infusions at t = 0 weeks and t = 6 weeks.
2. Autologous faecal infusions at t = 0 weeks and t = 6 weeks.

Randomly permuted blocks of size two and four will be used with no stratification. On the day of faecal transplantation, both patient and donors will deliver faeces produced that morning. Randomisation will be performed by one of the 'randomisation-assistants', who is designated to this task. To guarantee blinding, the randomisation-assistant will make sure the randomised treatment is not traceable to the donor or the patient. The blinded faeces will be brought to the Laboratory, where the preparation of the faeces will be done by one of the investigators. Detailed information about the preparation process is outlined under 'FMT procedure'. During the second FMT at 6 weeks, faeces will be processed according to the randomisation performed on the first transplantation day. The randomisation-assistant is the only person who will know which treatment the patient will be given and will have no role in further parts of the study. The randomisation list will be kept under secured access by Castor EDC. In case of an emergency, the study treatment can be unblinded after consultation of the principal investigator.

Intervention

FMT procedure

At baseline and at 6 weeks, patient and donor will collect a fresh morning stool sample in a small container and bring this to the AUMC for processing. Upon arrival of the patient in the hospital, a nasoduodenal tube will be positioned under direct imaging, with the Cortrak® electromagnetic sensing device.[35] After placement of the nasoduodenal tube, bowel lavage with 1.5–3.5 litres of macrogol electrolytes (Klean-Prep®) solution will be performed according to standard protocols to ensure complete bowel lavage. The amount of solution that is given depends on the rapidity by

Confidential: For Review Only

which the bowel is cleaned. Finally, a faecal suspension of 200cc will be infused in the duodenum of the patient through the nasoduodenal tube.

Preparation of faecal infusion product

On the day of infusion, a fresh faeces sample (100 – 200 g on average) of either the donor (allogeneic) or patient (autologous) will be used. Time of collection will be recorded. The faeces will be weighted and mixed with 200 – 400 ml Saline (0.9% NaCl) until fully homogenised. Next, the faeces solution is poured through a double gauze and debris of large size will be removed. This step will be repeated. Afterwards, the homogenised solution will be decanted through a metal funnel into a 200 cc sterile plastic bottle. All steps are performed under a fume-hood by one of the co-investigators. Within 6 hours after production by the donor, the faeces will be installed through the nasoduodenal tube in the patient

Outcomes

All below mentioned outcome measures apply to patients.

Primary outcome

The primary objective of this RCT is to assess the feasibility of our study protocol. This will be assessed by evaluating the process of patient recruitment and screening, the patient drop-out rate and the incidence rates of adverse events. Table 5 delineates the feasibility outcome measures, including measurement instruments and statistical analyses.

Secondary outcomes

Secondary objectives include the proportion of patients with > 50% reduction of their abdominal pain intensity and pain frequency compared to baseline at t=12 weeks after the first FMT. This will be assessed with the pain component of the IBS-SSS.[34] Table 5 also describes all secondary outcome measures.

Confidential: For Review Only

Table 5. Trial outcome measures, instruments and analyses

	Outcome measures	Instrument	Statistical analysis
Feasibility outcomes	**Patient recruitment**	**Patient recruitment per month**	1 patient/month recruited
	**Patient screening**	**Patient eligibility**	> 80 % of patients
	**Patient drop-out**	**Patient drop-out rate after randomization**<br>Including patient acceptance to accomplish repetitive faecal microbiota transplantations (FMTs)	< 20% of patients
	**Serious adverse events related to FMT**	**Hospitalization or increase of > 100 points on pain component of IBS-SSS**	< 10% of patients
	**Stool sample collection**	**Patients provide all necessary stool samples**<br>Morning stool samples will be collected during all study visits	> 90% of the required samples
Efficacy outcomes	**> 50% reduction of abdominal pain intensity and pain frequency compared to baseline at 12 (T3), 24 (T5) and 48 (T6) weeks after first FMT**	**Pain component of Irritable Bowel Syndrome Severity Scoring System (IBS-SSS) score**[34]<br>With two questions, the severity and frequency of the abdominal pain on the last 10 days is measured. The IBS-SSS is the only symptom severity scale that has been responsive to treatment effects.[36] It has been recommended as a good instrument to obtain information on specific IBS related symptoms.[37]	Fisher's exact or chi-square to compare proportions
	**Change in gut microbiota composition**	**MiSeq Illumina Sequencing**<br>Morning stool samples will be collected to profile the faecal microbiota composition by sequencing of the V4 region of the 16S ribosomal RNA (rRNA) gene	- Alfa-diversity (OTU count for species richness, Shannon index for species diversity)<br>- Beta-diversity (Bray-Curtis dissimilarity for microbial abundances)<br>- UniFrac distance
	**Change in gut mycobiome composition**	**ITS sequencing**<br>Morning stool sample will be collected to profile the faecal mycobiome composition by high-throughput rDNA sequencing of fungal internal transcribed spacer (ITS)-1 regions	- RDP-II Naïve Bayesian Classifier<br>- Bray-Curtis dissimilarity Index<br>- One-way permutational multivariate analysis of variance (PERMANOVA)
	**Change in gut metabolome composition**	**Capillary electrophoresis time-of-flight mass spectrometry (CE-TOF-MS)**<br>Morning stool sample will be collected to profile the faecal metabolome composition by CE-TOF-MS	- Spearman's correlation coefficient<br>- Wilcoxon's signed-rank test<br>- PCoA
	**Number of adverse events**	**Patient CRF**	Fisher's exact or chi-square to compare proportions

	**Number of rescue medication**	**Patient CRF**	Fisher's exact or chi-square to compare proportions
	**Total IBS-SSS score**	**Irritable Bowel Syndrome Severity Scoring System (IBS-SSS) score**[34] The IBS-SSS is the only symptom severity scale that has been responsive to treatment effects.[36] It has been recommended as a good instrument to obtain information on specific IBS related symptoms.[37]	Student's independent t-test or Mann Whitney U-test
	**Health related quality of life**	**Irritable Bowel Syndrome – Quality of Life (IBS-QOL) questionnaire**[38] This questionnaire is a 34-item assessment of the degree to which the IBS interferes with patient quality of life and consists of eight domains: dysphoria, interference with activities, body image, health worry, food avoidance, social reactions, sexual health, and effect on relationships.[38]	Student's independent t-test or Mann Whitney U-test
	**Generic quality of life**	**Medical Outcomes Study 36-item Short Form Health Survey (SF-36)** The SF-36 questionnaire consists of 36 questions regarding eight dimensions of health perception: limitations in physical functioning, role limitation due to physical health problems, bodily pain, general health perception, vitality, social functioning, role limitations due to emotional limitations, and mental health. A score between 0 (worst possible quality of life) and 100 (best possible quality of life) can be obtained. The reliability has been proven extensively for diverse patient groups and it is validated for the Dutch population.[39] The SF-36 is described as adequate for persons 14 years of age and older.[40]	Student's independent t-test or Mann Whitney U-test
	**Depression and anxiety**	**Hospital Anxiety and Depression Scale (HADS)** The HADS is divided into two 7-item scales, with answers on a four-point scale (0-3). Higher scores indicate a higher level of anxiety or depression (range 0-21). A scale score of ≥8 (cut-off score) indicates clinically significant anxiety or depression. The Dutch version of the HADS showed satisfactory validity and reliability.[41]	Student's independent t-test or Mann Whitney U-test
	**Absence of school or work, healthcare resources and costs**	**Adapted version of the Dutch Health and Labor Questionnaire**[42] School or work absenteeism and indirect healthcare utilization costs are measures by three items. Adolescents indicate whether they have been absent from school or work due to abdominal pain complaints, and if yes, the amount of hours per week.<br>For the indirect costs of healthcare utilization, adolescents indicate additional	Student's independent t-test or Mann Whitney U-test

	costs they had due to symptoms of abdominal pain over the past 4 weeks.	
**Impact of treatment**	**Adapted version of the Patient Satisfaction and Preference Questionnaire**[43]<br>Impact of FMT treatment will be assessed using 5 questions, which are based on the Patient Satisfaction and Preference Questionnaire used in another RCT on FMT in patients with recurrent Clostridium Difficile infection.[43] The questions address thoughts on how unpleasant and how dirty participants find the idea of getting a faecal transplant.	Student's independent t-test or Mann Whitney U-test
**Adequate relief**	**One question:**<br>''Did you have adequate relief of IBS symptoms (abdominal discomfort/pain, bowel habits, and other symptoms like nausea and bloating) over the past week?'' (Yes/No)	Fisher's exact or chi-square to compare proportions
**Plasma biomarkers**:<br>− Intestinal fatty acid-binding protein (I-FABP)<br>− Smooth muscle protein of 22 kDa (SM-22)<br>− Citrulline	**Vena puncture**<br>EDTA vacuum tubes were used. All blood samples were centrifuged at 4000 rpm, at 41C for 15 minutes to obtain plasma. Plasma was immediately stored in aliquots at -80°C until analysis	Student's independent t-test or Mann Whitney U-test
**Safety parameters**<br>− C-reactive Protein (CRP)<br>− Liver function<br>− Renal function	**Vena puncture**<br>EDTA vacuum tubes were used. All blood samples were centrifuged at 4000 rpm, at 41C for 15 minutes to obtain plasma. Plasma was immediately stored in aliquots at -80°C until analysis	Student's independent t-test or Mann Whitney U-test
**Dietary intake**	**Dietary diary**<br>Dietary intake lists are filled out 7 days prior to each faecal sample collection.	Fisher's exact or chi-square to compare proportions

CE-TOF-MS = capillary electrophoresis time-of-flight mass spectrometry; CRF = case report form; CRP = c-reactive protein; EDTA = ethylenediaminetetraacetic acid; FMT = faecal microbiota transplantation; HADS = Hospital Anxiety and Depression Scale; I-FABP = intestinal fatty acid-binding protein; IBS = irritable bowel syndrome; IBS-QOL = irritable bowel syndrome – quality of life; IBS-SSS = irritable bowel syndrome – severity scoring system; ITS = internal transcribed spacer; OTU = operational taxonomic unit; PcOA = principal coordinate analysis; RCT = randomized controlled trial; RDP = Ribosomal Database Project; rRNA = ribosomal RNA; SF-36 = Study 36-item Short Form Health Survey; SM-22 = smooth muscle protein of 22 kDa

Participant timeline

Figure 1 displays the time schedule of enrolment, interventions, assessments and visits for participating patients.

Sample size calculation

Since this is a pilot study, a reliable sample size calculation is not feasible. In accordance with recruitment recommendations,[44,45] a minimum of 15 patients per treatment group will be included. In addition, based on accumulated evidence with 16S rRNA sequencing using MiSeq, Illumina Platform, a sample size of 20 individuals is normally enough to detect relevant differences in the microbiota. Hence, a total sample size of N=30 seems adequate. In order to reduce heterogeneity in faecal transplants, one donor will donate faeces to approximately 3 patients, which implicates that 5 donors are needed for 30 patients.

Statistical analysis

All primary and secondary outcomes will be analysed according to the intention to treat (ITT) analysis. See Table 5 for statistical analyses of primary and secondary outcome measures. Significance is set at $\alpha = 0.05$ for all analyses.

Monitoring

Data monitoring

In order to optimise safety of the study during inclusion, patient data will be disclosed to a data safety monitoring board (DSMB) when 50% of the intended sample size is attained and has reached 12 weeks follow up. The advice(s) of the DSMB will be notified upon receipt by the sponsor to the METC that approved the protocol. With this notification a statement will be included indicating whether the advice will be followed.

Harms

https://mc.manuscriptcentral.com/bmjpo

Confidential: For Review Only

The risks associated with participation in this RCT can be considered moderate, because of the minimal invasive treatment. Nasoduodenal tube positioning through a Cortrak® electromagnetic sensing device carries a little risk of complications like aspiration, perforation or mal-positioning. If there is any doubt of malposition of the tube, a plain abdominal X-ray will be performed. To prevent complications, patients with swallowing disorders will not be included in this study.

Recent meta-analyses on clinical outcomes of FMT in general concluded that no serious adverse events were attributable to FMT.[30,46] Adverse events (AEs) were infrequent and mostly self-limiting (i.e. diarrhoea, abdominal distension, nausea and vomiting) and no differences existed in the number of AEs between donor FMT and control patients.[30,46] In our study, AEs will be monitored throughout the whole study. In order to make the risk for transmission of infectious diseases as small as possible rescreening of the faecal donors will be performed according to Table 6. In accordance to the legal requirements in the Netherlands (article 10, subsection 1, WMO), the investigator will inform the subjects and the reviewing accredited METC if harmful events occur. When there are indications that the disadvantage of participation may be significantly greater than was described in the research proposal, the study will be suspended pending a further positive decision by the accredited METC. The investigator will take care that all subjects are kept informed.

Confidential: For Review Only

Table 6. Time interval of donor rescreening*				
	Rescreening interval			
	Pre-FMT	***4 weeks***	***8 weeks***	***26 weeks***
Short rescreening questionnaire	x			
Extensive rescreening questionnaire				x
**Faeces screening**				
Calprotectine				x
Bacteria				x
Antibiotic Resistant Bacteria			x	
Viruses				x
Parasites				x
Non-pathogenic parasites**				x
Other				x
**Serum screening**				
Hematology				x
Bacteria				x
Viruses				x
Cytomegalovirus (CMV) *Epstein-Barr Virus (EBV)*		x*		
Parasites				x

* for specification of screening items see Table 4: specification of donor screening
** when a donor is seronegative for EBV IgG and/or CMV IgG

Ethics and dissemination

This study was approved by the Medical Ethics Research Committee of the AUMC in Amsterdam, the Netherlands. All important protocol amendments will be presented to the Medical Ethics Committee of the AUMC and will await approval before they are implemented.

Review Only

DISCUSSION AND CONCLUSION

IBS is a chronic and disabling condition, which can pose great impact on daily life of patients, reflected in decreased quality of life,[4,5] high work or school absence,[6,7] a higher risk to develop depressive and anxiety disorders.[7,8] and substantial healthcare costs.[9,10] Effective management strategies for adolescents and adults in the form of antidepressants, peppermint oil, cognitive behavioural therapy, hypnotherapy, probiotics and low FODMAP diet exist. However, a subgroup of IBS patients remains symptomatic. New effective treatment options for this subgroup are warranted and might be targeted on the altered microbiome in IBS patients.[24]

Up to now, six RCTs have been performed to assess the effect of FMT in IBS in adults. Two trials assessed the effect of FMT administered by capsules, two evaluated the effect of FMT delivered by colonoscopy, one via gastroscope, and one by nasojejunal tube.[31] It appears that the efficacy of FMT is associated with the methodology of FMT and placebo, as donor faeces administered by colonoscopy, gastroscopy or nasojejunal tube demonstrated a clinically significant improvement in global IBS symptoms in comparison with autologous FMT via the same route, whereas stool capsules did not demonstrate any beneficial effect compared to placebo capsules.[31]

The present pilot study is the first to assess the feasibility of FMT in adolescents with refractory IBS according to the Rome IV criteria. Furthermore, the efficacy of FMT on abdominal pain symptoms in these patients is explored. By designing this specific treatment protocol, a unique opportunity is created to investigate potential beneficial effects of restoring the gut microbiota composition on abdominal pain complaints. Data of this study will help determine optimal study conditions and inform the choice of endpoints for future, larger size, double-blind RCTs on FMT in adolescents with IBS. Furthermore, this study will define preliminary efficacy results of the use of FMT in these patients. In addition, this study will enable us to analyse in detail which microbiota components might predict a positive response to FMT.

Confidential: For Review Only

Our study has several strengths. First, the FMT will be administered via a nasoduodenal tube and it will be performed twice, since it has been demonstrated that this might enhance the effect of the FMT.[48] Another strength is the one-year follow-up, which allows us to assess the long-term effect of FMT.

A limitation of our study is the small sample size, which allows us to only encounter major effects of the FMT treatment. Furthermore, we decided to include IBS patients regardless of subtype, leading to a heterogeneous patient population which may affect the efficacy results. Moreover, it is unclear what the effect of bowel lavage is on the efficacy of FMT and on microbiome composition. Studies with and without bowel preparations before FMT demonstrate great efficacy.[48,49] In addition, it has been shown that bowel preparation can disrupt the colonic ecosystem were the overall microbiome composition recovers to baseline within 14 days after bowel cleansing.[50] Our efficacy outcome measure is assessed at 12 weeks after the first FMT (and 6 weeks after the second FMT), which minimises the effect that the bowel cleansing can have on the microbiome composition.

In conclusion, the results of this trial will provide preliminary evidence for the use of FMT in adolescents with refractory IBS. The results will inform future larger, double-blind, placebo-controlled trials on the right sample size, on the feasibility of this study design, on efficacy outcome measures and on the potential of the microbiome to be a therapeutic target in IBS.

Confidential: For Review Only

Contributorship Statement

MAB is the principal investigator, designed the study, wrote the protocol, supervised the trial, and supervised drafting of the manuscript. JZ participated in the design of the study, wrote the protocol, coordinated part of the trial, and was responsible for data collection, analysis and drafting the manuscript. CMAB coordinates the trial, and is responsible for data collection, analysis and drafting the manuscript. AV and MN contributed to the design of the trial, critically revised the protocol, and supervised drafting of the manuscript.

Funding

This research received no specific grant from any funding agency in the public, commercial or not-for-profit sectors.

Competing interests

M. Nieuwdorp is in the scientific advisory board of Caelus Health and Kaleido BioSciences; however, none of these are directly related to the current manuscript.

The other have no competing interests relevant to this article to disclose.

Acknowledgements

Dr. I.J.N. Koppen and dr. D.R. Hoekman provided substantial conceptual contributions. Prof. dr. A.H. Zwinderman provided contributions to statistical considerations for the trial.

Confidential: For Review Only

REFERENCES

1 Sperber AD, Dumitrascu D, Fukudo S, *et al.* The global prevalence of IBS in adults remains elusive due to the heterogeneity of studies: a Rome Foundation working team literature review. *Gut* 2017;**66**:1075–82. doi:10.1136/gutjnl-2015-311240

2 Korterink JJ, Diederen K, Benninga MA, *et al.* Epidemiology of pediatric functional abdominal pain disorders: a meta-analysis. *PLoS One* 2015;**10**:e0126982. doi:10.1371/journal.pone.0126982

3 Sagawa T, Okamura S, Kakizaki S, *et al.* Functional gastrointestinal disorders in adolescents and quality of school life. *J Gastroenterol Hepatol* 2013;**28**:285–90. doi:10.1111/j.1440-1746.2012.07257.x

4 Gralnek IM, Hays RD, Kilbourne A, *et al.* The impact of irritable bowel syndrome on health-related quality of life. *Gastroenterology* 2000;**119**:654–60.

5 Youssef NN. Quality of Life for Children With Functional Abdominal Pain: A Comparison Study of Patients' and Parents' Perceptions. *Pediatrics* 2006;**117**:54–9. doi:10.1542/peds.2005-0114

6 Canavan C, West J, Card T. Review article: the economic impact of the irritable bowel syndrome. *Aliment Pharmacol Ther* 2014;**40**:1023–34. doi:10.1111/apt.12938

7 Youssef NN, Atienza K, Langseder AL, *et al.* Chronic abdominal pain and depressive symptoms: analysis of the national longitudinal study of adolescent health. *Clin Gastroenterol Hepatol* 2008;**6**:329–32. doi:10.1016/j.cgh.2007.12.019

8 Fond G, Loundou A, Hamdani N, *et al.* Anxiety and depression comorbidities in irritable bowel syndrome (IBS): a systematic review and meta-analysis. *Eur Arch Psychiatry Clin Neurosci* 2014;**264**:651–60. doi:10.1007/s00406-014-0502-z

9 Brandt LJ, Chey WD, Foxx-Orenstein AE, *et al.* An Evidence-Based Position Statement on the Management of Irritable Bowel Syndrome. *Am J Gastroenterol* 2008;**104**:S1–35. doi:10.1038/ajg.2008.122

10 Hoekman DR, Rutten JMTM, Vlieger AM, *et al.* Annual Costs of Care for Pediatric Irritable Bowel Syndrome, Functional Abdominal Pain, and Functional Abdominal Pain Syndrome. *J Pediatr* 2015;**167**:1103–8.e2. doi:10.1016/j.jpeds.2015.07.058

11 Hyams JS, Di Lorenzo C, Saps M, *et al.* Childhood Functional Gastrointestinal Disorders: Child/Adolescent. *Gastroenterology* 2016;**150**:1456–68.e2. doi:10.1053/j.gastro.2016.02.015

12 Drossman DA. The functional gastrointestinal disorders and the Rome III process. *Gastroenterology* 2006;**130**:1377–90. doi:10.1053/j.gastro.2006.03.008

13 Rutten JMTM, Korterink JJ, Venmans LMAJ, *et al.* [Guideline on functional abdominal pain in children]. *Ned Tijdschr Geneeskd* 2017;**161**:D781.

14 Ford AC, Lacy BE, Harris LA, *et al.* Effect of Antidepressants and Psychological Therapies in Irritable Bowel Syndrome. *Am J Gastroenterol* 2019;**114**:21–39. doi:10.1038/s41395-018-0222-5

15 Black CJ, Yuan Y, Selinger CP, *et al.* Efficacy of soluble fibre, antispasmodic drugs, and gut–brain neuromodulators in irritable bowel syndrome: a systematic review and network meta-analysis. *Lancet Gastroenterol Hepatol* Published Online First: 16 December 2019.

Confidential: For Review Only

doi:10.1016/S2468-1253(19)30324-3

16 Martin AE, Newlove-Delgado T V, Abbott RA, *et al.* Pharmacological interventions for recurrent abdominal pain in childhood. In: Martin AE, ed. *Cochrane Database of Systematic Reviews*. Chichester, UK: : John Wiley & Sons, Ltd 2017. CD010973. doi:10.1002/14651858.CD010973.pub2

17 Ford AC, Harris LA, Lacy BE, *et al.* Systematic review with meta-analysis: the efficacy of prebiotics, probiotics, synbiotics and antibiotics in irritable bowel syndrome. *Aliment Pharmacol Ther* 2018;**48**:1044–60. doi:10.1111/apt.15001

18 Newlove-Delgado T V, Martin AE, Abbott RA, *et al.* Dietary interventions for recurrent abdominal pain in childhood. *Cochrane database Syst Rev* 2017;**3**:CD010972. doi:10.1002/14651858.CD010972.pub2

19 Dionne J, Ford AC, Yuan Y, *et al.* A Systematic Review and Meta-Analysis Evaluating the Efficacy of a Gluten-Free Diet and a Low FODMAPS Diet in Treating Symptoms of Irritable Bowel Syndrome. *Am J Gastroenterol* 2018;**113**:1290–300. doi:10.1038/s41395-018-0195-4

20 Abbott RA, Martin AE, Newlove-Delgado T V, *et al.* Psychosocial interventions for recurrent abdominal pain in childhood. *Cochrane database Syst Rev* 2017;**1**:CD010971. doi:10.1002/14651858.CD010971.pub2

21 Vlieger AM, Rutten JMTM, Govers AMAP, *et al.* Long-term follow-up of gut-directed hypnotherapy vs. standard care in children with functional abdominal pain or irritable bowel syndrome. *Am J Gastroenterol* 2012;**107**:627–31. doi:10.1038/ajg.2011.487

22 Saulnier DM, Riehle K, Mistretta T-A, *et al.* Gastrointestinal microbiome signatures of pediatric patients with irritable bowel syndrome. *Gastroenterology* 2011;**141**:1782–91. doi:10.1053/j.gastro.2011.06.072

23 Tap J, Derrien M, Törnblom H, *et al.* Identification of an Intestinal Microbiota Signature Associated With Severity of Irritable Bowel Syndrome. *Gastroenterology* 2017;**152**:111–23.e8. doi:10.1053/j.gastro.2016.09.049

24 Pittayanon R, Lau JT, Yuan Y, *et al.* Gut Microbiota in Patients With Irritable Bowel Syndrome—A Systematic Review. *Gastroenterology* 2019;**157**:97–108. doi:10.1053/j.gastro.2019.03.049

25 Hyland NP, Quigley EMM, Brint E. Microbiota-host interactions in irritable bowel syndrome: epithelial barrier, immune regulation and brain-gut interactions. *World J Gastroenterol* 2014;**20**:8859–66. doi:10.3748/wjg.v20.i27.8859

26 Öhman L, Törnblom H, Simrén M. Crosstalk at the mucosal border: importance of the gut microenvironment in IBS. *Nat Rev Gastroenterol Hepatol* 2015;**12**:36–49. doi:10.1038/nrgastro.2014.200

27 van Nood E, Vrieze A, Nieuwdorp M, *et al.* Duodenal infusion of donor feces for recurrent Clostridium difficile. *N Engl J Med* 2013;**368**:407–15. doi:10.1056/NEJMoa1205037

28 Costello SP, Hughes PA, Waters O, *et al.* Effect of Fecal Microbiota Transplantation on 8-Week Remission in Patients With Ulcerative Colitis. *JAMA* 2019;**321**:156. doi:10.1001/jama.2018.20046

29 Kootte RS, Levin E, Salojärvi J, *et al.* Improvement of Insulin Sensitivity after Lean Donor Feces

Confidential: For Review Only

in Metabolic Syndrome Is Driven by Baseline Intestinal Microbiota Composition. *Cell Metab* 2017;**26**:611–9.e6. doi:10.1016/j.cmet.2017.09.008

30 Xu D, Chen VL, Steiner CA, *et al.* Efficacy of Fecal Microbiota Transplantation in Irritable Bowel Syndrome. *Am J Gastroenterol* 2019;**114**:1043–50. doi:10.14309/ajg.0000000000000198

31 Ianiro G, Eusebi LH, Black CJ, *et al.* Systematic review with meta-analysis: efficacy of faecal microbiota transplantation for the treatment of irritable bowel syndrome. *Aliment Pharmacol Ther* 2019;**50**:240–8. doi:10.1111/apt.15330

32 El-Salhy M, Hatlebakk JG, Gilja OH, *et al.* Efficacy of faecal microbiota transplantation for patients with irritable bowel syndrome in a randomised, double-blind, placebo-controlled study. *Gut* 2019;:gutjnl – 2019–319630. doi:10.1136/gutjnl-2019-319630

33 Terveer EM, van Beurden YH, Goorhuis A, *et al.* How to: Establish and run a stool bank. *Clin Microbiol Infect* 2017;**23**:924–30. doi:10.1016/j.cmi.2017.05.015

34 Francis CY, Morris J, Whorwell PJ. The irritable bowel severity scoring system: a simple method of monitoring irritable bowel syndrome and its progress. *Aliment Pharmacol Ther* 1997;**11**:395–402.

35 Rao MM, Kallam R, Flindall I, *et al.* Use of Cortrak--an electromagnetic sensing device in placement of enteral feeding tubes. *Proc Nutr Soc* 2008;**67**:E109. doi:10.1017/S0029665108007416

36 Irvine EJ, Tack J, Crowell MD, *et al.* Design of Treatment Trials for Functional Gastrointestinal Disorders. *Gastroenterology* 2016;**150**:1469–80.e1. doi:10.1053/j.gastro.2016.02.010

37 Bijkerk CJ, de Wit NJ, Muris JWM, *et al.* Outcome measures in irritable bowel syndrome: comparison of psychometric and methodological characteristics. *Am J Gastroenterol* 2003;**98**:122–7. doi:10.1111/j.1572-0241.2003.07158.x

38 Patrick DL, Drossman DA, Frederick IO, *et al.* Quality of life in persons with irritable bowel syndrome: development and validation of a new measure. *Dig Dis Sci* 1998;**43**:400–11.

39 Aaronson NK, Muller M, Cohen PD, *et al.* Translation, validation, and norming of the Dutch language version of the SF-36 Health Survey in community and chronic disease populations. *J Clin Epidemiol* 1998;**51**:1055–68.

40 Ware JE. SF-36 health survey update. *Spine (Phila Pa 1976)* 2000;**25**:3130–9.

41 Spinhoven P, Ormel J, Sloekers PP, *et al.* A validation study of the Hospital Anxiety and Depression Scale (HADS) in different groups of Dutch subjects. *Psychol Med* 1997;**27**:363–70.

42 Van Roijen L, Essink-bot M-L, Koopmanschap MA, *et al.* Labor and Health Status in Economic Evaluation of Health Care: The Health and Labor Questionnaire. *Int J Technol Assess Health Care* 1996;**12**:405–15. doi:10.1017/S0266462300009764

43 Kao D, Roach B, Silva M, *et al.* Effect of Oral Capsule– vs Colonoscopy-Delivered Fecal Microbiota Transplantation on Recurrent *Clostridium difficile* Infection. *JAMA* 2017;**318**:1985. doi:10.1001/jama.2017.17077

44 Sim J, Lewis M. The size of a pilot study for a clinical trial should be calculated in relation to considerations of precision and efficiency. *J Clin Epidemiol* 2012;**65**:301–8. doi:10.1016/j.jclinepi.2011.07.011

Confidential: For Review Only

45 Lancaster GA, Dodd S, Williamson PR. Design and analysis of pilot studies: recommendations for good practice. *J Eval Clin Pract* 2004;**10**:307–12. doi:10.1111/j.2002.384.doc.x

46 Lai CY, Sung J, Cheng F, *et al.* Systematic review with meta-analysis: review of donor features, procedures and outcomes in 168 clinical studies of faecal microbiota transplantation. *Aliment Pharmacol Ther* 2019;**49**:354–63. doi:10.1111/apt.15116

47 Information Pertaining to Additional Safety Protections Regarding Use of Fecal Microbiota for Transplantation – Screening and Testing of Stool Donors for Multi-drug Resistant Organisms | FDA. https://www.fda.gov/vaccines-blood-biologics/safety-availability-biologics/information-pertaining-additional-safety-protections-regarding-use-fecal-microbiota-transplantation (accessed 21 Oct 2019).

48 Ianiro G, Maida M, Burisch J, *et al.* Efficacy of different faecal microbiota transplantation protocols for Clostridium difficile infection: A systematic review and meta-analysis. *United Eur Gastroenterol J* 2018;**6**:1232–44. doi:10.1177/2050640618780762

49 Postigo R, Kim JH. Colonoscopic versus nasogastric fecal transplantation for the treatment of Clostridium difficile infection: a review and pooled analysis. *Infection* 2012;**40**:643–8. doi:10.1007/s15010-012-0307-9

50 Nagata N, Tohya M, Fukuda S, *et al.* Effects of bowel preparation on the human gut microbiome and metabolome. *Sci Rep* 2019;**9**:4042. doi:10.1038/s41598-019-40182-9

Confidential: For Review Only

Address correspondence

Judith Zeevenhooven, Emma Children's Hospital, Amsterdam UMC, University of Amsterdam, Paediatric Gastroenterology, Room C2-312, PO Box 22700, 1100 DD Amsterdam, The Netherlands.

Telephone: +3120-5662906. Email: j.zeevenhooven@amsterdamumc.nl

Confidential: For Review Only

Figure legends

Figure 1. Trial design. After adolescents sign the informed consent form (T-2), patients complete the baseline pain diary, the IBS-SSS and deliver stool samples and blood samples for eligibility screening. At T0, adolescents are randomised in the allogeneic or autologous FMT group.

Confidential: For Review Only

	Enrolment	Allocation	During treatment		Follow-up, *Number of weeks after first FMT*			
**TIMEPOINT**	**T -2**	**T0**	**T1**	**T2**	**T3**	**T4**	**T5**	**T6**
	**-2 weeks**	**Baseline**	**3 weeks**	**6 weeks**	**12 weeks**	**16 weeks**	**24 weeks**	**48 weeks**
	**Screening patient**	**First FMT**		**Second FMT**				
**ENROLMENT:**								
AMC visit	X	X		X	X		X	X
Phone assessment			X			X		
Eligibility screen	X							
Informed consent	X							
Allocation		X						
**INTERVENTIONS:**								
Allogeneic FMT		↔		↔				
Autologous FMT		↔		↔				
**ASSESSMENTS:**								
Pain diary card	X							
IBS-SSS	X	X	X	X	X	X	X	X
Morning stool sample	X	X		X	X		X	X
Blood samples (20 ml)	X	X		X	X			
Adverse events			X	X	X	X	X	X
Questionnaires:<br>- Quality of life<br>- Depression/anxiety<br>- School/work absenteeism<br>- Impact of treatment<br>- Adequate relief		X		X	X		X	X
Dietary booklet	X	X		X	X		X	X

Figure 1. Trial design

After adolescents sign the informed consent form (T-2), patients complete the baseline pain diary, the IBS-SSS and deliver stool samples and blood samples for eligibility screening. At T0, adolescents are randomised in the allogeneic or autologous FMT group.

FMT = fecal microbiota transplantation; IBS-SSS = Irritable Bowel Syndrome Severity Scoring System

Confidential: For Review Only

Supplementary Table 1. Exclusion criteria patients and donors
**Patients**
Use of systemic antibiotics in preceding 6 weeks
Use of probiotic treatment in preceding 6 weeks
Use of concomitant medication, including proton pomp inhibitors (PPI) and vasopressine medication. Pain medication in the form of Paracetamol or NSAIDs is allowed.
Current use of drugs which influence gastrointestinal motility (erythromycin, azithromycin, butyl scopolamine, domperidone, peppermint oil capsules, iberogast)
Current treatment by another health care professional for abdominal symptoms
Current treatment by psychologist or shrink for known anxiety or depression disorder
Known swallowing disorder
Known diagnosis of inflammatory bowel disease (i.e. Crohn's disease or ulcerative colitis)
Known concomitant organic gastrointestinal disease
Known diagnosis of an autoimmune disease (e.g. hypo- or hyperthyroidism, celiac disease, rheumatoid arthritis)
Condition leading to profound immunosuppression (HIV, infectious diseases leading to immunosuppression, bone marrow malignancies)
Known diagnosis of cystic fibrosis
Known diagnosis of porphyria
Known pregnancy or current lactation
Use of systematic chemotherapy
Life expectancy < 12 months
Current Intensive Care Unit-stay
XTC, amphetamine or cocaine abuse
Known intra-abdominal fistula
Signs of ileus, diminished passage
Allergy to macrogol or substituents, e.g. peanuts, shellfish
History of surgery:<br>o *Hemicolectomy (defined as: surgery resulting in a resection of > 0.5 of the colon)*<br>o *Presence of a pouch due to surgery*<br>o *Presence of stoma*
Insufficient knowledge of the Dutch language
**Donors**
Abnormal bowel motions, abdominal complaints or symptoms indicative of irritable bowel syndrome
An extensive travel behaviour
Higher risk of colonization with multidrug- resistant organisms including:<br>o *Health care workers*<br>o *Persons who have recently been hospitalized or discharged from long term care facilities*<br>o *Persons who regularly attend outpatient medical or surgical clinics*<br>o *Persons who have recently engaged in medical tourism*
Unsafe sex practice (assessed with standardized questionnaire)
Use of any medication including PPI
Antibiotic treatment in the past 12 weeks
A positive history/clinical evidence for inflammatory bowel disease (i.e. Crohn's disease or ulcerative colitis)
A positive history/clinical evidence for other gastrointestinal diseases, including chronic diarrhoea or chronic constipation

Patients receiving immunosuppressive medications or a positive history/clinical evidence for autoimmune disease including:<br>o *Type 1 diabetes*<br>o *Hashimoto hypothyroidism*<br>o *Graves hyperthyroidism*<br>o *Rheumatoid arthritis*<br>o *Celiac disease*
History of or present known malignant disease and/or patients who are receiving systemic anti-neoplastic agents
Known psychiatric disease (i.e. depression, schizophrenia, autism, Asperger's syndrome)
Known chronic neurological/neurodegenerative disease (e.g. Parkinson's disease, multiple sclerosis)
Positive blood tests for the presence of: HIV, HTLV, lues, Strongyloides
Active hepatitis A, B-, C- or E-virus infection or known exposure within recent 12 months
Acute infection with cytomegalovirus (CMV) or Epstein-Barr virus (EBV)
Chronic pain syndromes (e.g. fibromyalgia)
Major relevant allergies (e.g. food allergy, multiple allergies)
Recent (gastrointestinal) infection within last 6 months
Tattoo or body piercing placement within last 6 months
Alcohol abuse (>3 units/day)
Known risk of Creutzfeldt Jacob's disease
History of current use of IV drugs
History of treatment with growth factors

For Review Only

BMJ Paediatrics Open

Protocol for a pilot randomised, double-blind, placebo-controlled trial for assessing the feasibility and efficacy of faecal microbiota transplantation in adolescents with refractory irritable bowel syndrome: FAIS Trial

Journal:	*BMJ Paediatrics Open*
Manuscript ID	bmjpo-2020-000689.R1
Article Type:	Protocol
Date Submitted by the Author:	30-Jun-2020
Complete List of Authors:	Zeevenhooven, Judith; Emma Childrens Hospital AMC, Paediatric Gastroenterology<br>de Bruijn, Clara; Emma Childrens Hospital AMC, Paediatric Gastroenterology<br>Vlieger, Arine; Sint Antonius Hospital, Department of Paediatrics<br>Nieuwdorp, Max; Amsterdam UMC - Locatie AMC, Department of Internal Medicine<br>Benninga, Marc; Emma Kinderziekenhuis AMC, Paediatric Gastroenterology and Nutrition
Keywords:	Gastroenterology

SCHOLARONE™
Manuscripts

I, the Submitting Author has the right to grant and does grant on behalf of all authors of the Work (as defined in the below author licence), an exclusive licence and/or a non-exclusive licence for contributions from authors who are: i) UK Crown employees; ii) where BMJ has agreed a CC-BY licence shall apply, and/or iii) in accordance with the terms applicable for US Federal Government officers or employees acting as part of their official duties; on a worldwide, perpetual, irrevocable, royalty-free basis to BMJ Publishing Group Ltd ("BMJ") its licensees and where the relevant Journal is co-owned by BMJ to the co-owners of the Journal, to publish the Work in this journal and any other BMJ products and to exploit all rights, as set out in our licence.

The Submitting Author accepts and understands that any supply made under these terms is made by BMJ to the Submitting Author unless you are acting as an employee on behalf of your employer or a postgraduate student of an affiliated institution which is paying any applicable article publishing charge ("APC") for Open Access articles. Where the Submitting Author wishes to make the Work available on an Open Access basis (and intends to pay the relevant APC), the terms of reuse of such Open Access shall be governed by a Creative Commons licence – details of these licences and which Creative Commons licence will apply to this Work are set out in our licence referred to above.

Other than as permitted in any relevant BMJ Author's Self Archiving Policies, I confirm this Work has not been accepted for publication elsewhere, is not being considered for publication elsewhere and does not duplicate material already published. I confirm all authors consent to publication of this Work and authorise the granting of this licence.

Confidential: For Review Only

Protocol for a pilot randomised, double-blind, placebo-controlled trial for assessing the feasibility and efficacy of faecal microbiota transplantation in adolescents with refractory irritable bowel syndrome: FAIS Trial

Authors

Judith Zeevenhooven[1], Clara M.A. de Bruijn[1], Arine M. Vlieger[2], Max Nieuwdorp[3], Marc A. Benninga[1]

Affiliations

1. Emma Children's Hospital, Amsterdam UMC, University of Amsterdam, Paediatric Gastroenterology, Hepatology and Nutrition, Amsterdam, The Netherlands
2. Department of Paediatrics, St. Antonius Hospital, Nieuwegein, The Netherlands
3. Department of Internal Medicine, Amsterdam UMC, University of Amsterdam

Address correspondence

Judith Zeevenhooven, Emma Children's Hospital, Amsterdam UMC, University of Amsterdam, Paediatric Gastroenterology, Room C2-312, PO Box 22700, 1100 DD Amsterdam, The Netherlands. Telephone: +3120-5662906. Email: j.zeevenhooven@amsterdamumc.nl

Word Count

Main text: 2503

Tables: 6

Figures: 1

Number of references: 50

Confidential: For Review Only

ABSTRACT

Background: Irritable bowel syndrome (IBS) is a common chronic medical condition, in both children and adults. Despite the availability of effective (non)pharmacological treatments, symptoms persist in a significant amount of IBS patients. Faecal microbiota transplantation (FMT) may be an effective alternative treatment in adolescents with refractory IBS through manipulation of the intestinal microbiota.

Methods and analysis: This randomised, placebo-controlled single-centre pilot study will assess feasibility and efficacy of FMT in 30 adolescents (16-21 years) with refractory IBS. Patients will be randomly allocated (1:1) to receive two allogeneic (healthy donor) or two autologous (own) faecal infusions at baseline and after 6 weeks. Primary outcomes will assess feasibility, including patient and donor recruitment, adherence, and incidence rates of adverse events. To evaluate clinical efficacy, secondary outcomes will include the proportion of patients with at least > 50% reduction of their abdominal pain intensity and frequency 12 weeks after the first FMT, and after 6 and 12 months follow-up. Other outcomes comprise changes in faecal gut microbiota composition, quality of life, depression and anxiety, school or work absenteeism and adequate relief, measured directly after FMTs and after 6 and 12 months of follow up.

Discussion: This RCT will investigate the feasibility and effectiveness of repetitive FMTs in adolescents with refractory IBS.

Ethics and dissemination: The study is approved by the Medical Research Ethics Committees AMC (MEC-AMC) in the Netherlands.

Trial registration details: Clinical trials registration number is NCT03074227.

Keywords: Faecal microbiota transplantation, FMT, irritable bowel syndrome, IBS, therapy resistant, adolescents

Confidential: For Review Only

What is known about the subject
➢ It is suggested that IBS symptoms are generated through an effect of the microbiome on the intestinal barrier, enteroendocrine system, the immune system and the gut-brain axis<br>➢ Faecal Microbiota Transplantation (FMT) administered via a nasoduodenal tube, is a new treatment regimen which modifies the gut microbiome through replacement of the patient microbiome by that of a healthy donor
**What this study hopes to add**
➢ This RCT will investigate the feasibility and effectiveness of repetitive FMTs in adolescents with refractory IBS<br>➢ This study will enable us to analyse in detail which microbiota components might predict a positive response to FMT

Confidential: For Review Only

BACKGROUND

Irritable bowel syndrome (IBS) according to the Rome IV criteria (Table 1) is a common chronic medical condition, with worldwide pooled prevalence rates in adults and children ranging from 5.8-17.5% and 6.2-11.9%, respectively.[1,2] Some studies report a peak prevalence in adolescents (12-19 years).[3] IBS impairs daily life, as patients report a decreased quality of life,[4,5] high work or school absence,[6,7] and a higher risk to develop depressive and anxiety disorders compared to healthy controls.[7,8] Consequently, healthcare costs are substantial.[9,10]

**Table 1. Rome IV criteria: Irritable Bowel Syndrome**[11]
**Diagnostic Criteria must include all of the following***
1. Abdominal pain at least 4 days per month associated with one or more of the following:<br>a. Related to defecation<br>b. A change in frequency of stool<br>c. A change in form (appearance) of stool
2. In children with constipation, the pain does not resolve with resolution of the constipation (children in whom the pain resolves have functional constipation, not irritable bowel syndrome)
3. After appropriate evaluation, the symptoms cannot be fully explained by another medical condition
**Criteria fulfilled for at least 2 months before diagnosis.*

Standard medical care for IBS consists of education, reassurance and simple dietary and behavioural advices.[12,13] Subsequently, either a pharmacological (tricyclic antidepressants (TCA), peppermint oil, linaclotide and lubiprostone) or non-pharmacological treatment (hypnotherapy and cognitive behavioural therapy) can be considered.[12–15] In the treatment of adolescent IBS patients, evidence for the efficacy of pharmacological agents is scarce and inconclusive.[16] In addition, some interventions that modify the microbiome, such as rifaximin or particular strains of probiotics, appear to have beneficial effects in IBS adult patients,[17] and in adolescent patients as well.[16,18] Some low quality evidence exists for the dietary low in fermentable oligo-, di- and monosaccharides and polyols (FODMAP) intervention in adult and adolescent IBS patients.[18,19] Finally, some psychological therapies, such as hypnotherapy, relaxation therapy and cognitive behavioural therapy are proven to be effective treatments for IBS.[14,20] Despite these available treatments, symptoms

Confidential: For Review Only

may persist in some IBS patients.[21] These IBS patients can be considered as therapy resistant (refractory) and might benefit from another potential treatment. Recent publications in children and adults indicate that altered gut microbiota may play an important role in the pathophysiology of IBS.[22–24] Symptoms may be generated through effects of the microbiome on the intestinal barrier, enteroendocrine system, the immune system, the gut-brain axis, regulation of bile acid deconjugation, but also via diet derived metabolites produced by the microbiota.[25,26] Therefore, manipulation of the intestinal microbiota by faecal microbiota transplantation (FMT), which modifies the gut microbiome through replacement of the patient microbiome by that of a healthy donor, in refractory IBS patients can potentially have beneficial effects on IBS symptoms. FMT has been shown to be highly effective in treating adults with recurrent *Clostridium difficile* infection [27] and yielded promising results in patients with ulcerative colitis [28] and metabolic syndrome.[29] For IBS, six randomized controlled trials (RCTs) on efficacy of FMT have been performed in adults.[30–32] Two recent meta-analyses on these trials concluded that FMT versus placebo yielded no significant improvement in IBS symptoms, but results were hampered by significant inconsistency due to important differences in FMT methodology.[30,31] To the best of our knowledge, no study has yet assessed the effect of FMT in adolescents with refractory IBS. Therefore, the objective of this RCT is to assess feasibility and effectiveness of FMT in adolescents with refractory IBS.

Confidential: For Review Only

METHODS

Trial design

The Faecal Administration in refractory Irritable bowel Syndrome (FAIS) trial is a double-blind, randomised, placebo-controlled single-centre pilot study. We aim to enrol 30 adolescents aged between 16 and 21 years, with refractory irritable bowel syndrome (Table 2). After randomisation, patients will either receive two allogenic faecal microbiota transplantations (FMTs) from a healthy donor or two autologous FMTs at baseline and after 6 weeks. The flow of the study protocol is presented in Figure 1.

Table 2. Refractory Irritable Bowel Syndrome
1. Irritable bowel syndrome (IBS) according to the Rome IV criteria
2. Symptoms are present for ≥ 12 months
3. Patients received adequate explanation, reassurance and dietary advice for their symptoms
4. There is an absence of response to a minimum of six sessions of psychological treatment, like hypnotherapy or cognitive behavioural therapy
5. There is an absence of response to an adequate dose of at least one pharmacological agent tried for a minimum of six weeks

Patient and public involvement

There was no involvement of patients or the public in the design of this RCT.

Procedure

Recruitment

<u>Patients</u>

Patients from the outpatient clinic of the Amsterdam University Medical Centre (AUMC) will be recruited by their treating gastroenterologist. Furthermore, patients from other hospitals can be referred to the AUMC for participation in this study. In addition, patients will be recruited throughout

Confidential: For Review Only

the Netherlands with help of online advertisement through IBS patient associations. Patient enrolment began in September 2018.

<u>Donors</u>

Healthy faecal donors will be recruited through advertisement in the form of posters, intranet network and emails, and via word by mouth.

Participant screening

<u>Patients</u>

Eligible patients will be invited for a screening visit. Informed consent from the participants will be obtained by the clinical research coordinator. During the screening visit, adolescents will undergo routine laboratory testing to exclude underlying organic disorders (Table 3). Furthermore, patients will fill out a pain diary.

**Table 3. Specification of patient screening**	
**Faeces screening**	
Calprotectine	
Bacteria	
Clostridium difficile	
Helicobacter pylori	
Parasites	
Giardia lamblia	Dientamoeba fragilis
Cryptosporidium spp.	Blastocystis hominis
Entamoeba histolytica	
Other	
Parasitic worm eggs	Protozoan Cysts and Oocysts
Larvae	
**Serum screening**	
Hematology	
Complete Blood Count (CBC)	Alkaline phosphatase (AF)
C-reactive protein (CRP)	Kreatinine
Bilirubine	Ureum
Aspartate aminotransferase (ASAT)	Estimated Glomerular Filtration Rate (EGFR)
Alanine aminotransferase (ALAT)	Anti-transglutaminase antibodies
Gamma-glutamyl transferase (GGT)	IgA
Viruses*	

Confidential: For Review

Cytomegalovirus (CMV)
Epstein-Barr Virus (EBV)
* In case of seronegativity, a matching seronegative donor will be used for FMT<br><br>AF = Alkaline phosphatase; ALAT = Alanine aminotransferase; ASAT = Aspartate aminotransferase; CBC = complete blood count; CMV = cytomegalovirus; CRP = C-reactive protein; EGFR = Estimated Glomerular Filtration Rate; EBV = Epstein-Barr virus; GGT = Gamma-glutamyl transferase;

<u>Donors</u>

Potential donors will be thoroughly screened according to the screenings protocol of the Netherlands Donor Faeces Bank (NDFB).[33] Potential donors have to complete an extensive questionnaire regarding risk factors for infectious diseases and factors potentially perturbing the intestinal microbiota. Exclusion criteria for donors are outlined under 'Eligibility criteria'. If donors are considered eligible after completing the questionnaire, they will undergo serum and faeces laboratory testing to exclude potentially transmittable diseases (Table 4).

Table 4. Specification of donor screening

**Faeces screening**	
Calprotectine	
Bacteria	
Clostridium difficile	Yersinia enterocolitica
Helicobacter pylori	Plesiomonas shigelloides
Salmonella spp	Pathogenic Campylobacter Spp.
Shigella spp.	Shiga toxin-producing Escherichia coli (STEC)
Antibiotic Resistant Bacteria	
Vancomycin-resistant Enterococcus (VRE)	Multidrug-resistant Gram-negative (MRGN) 3
Carbapenem-resistant Enterobacteriaceae (CRE)	MRGN 4
Methicillin-resistant Staphylococcus aureus (MRSA)	Extended spectrum beta-lactamase (ESBL)-producing Enterobactereacceae
Viruses	
Hepatitis E	Rotavirus
Norovirus Type I and II	Enterovirus
Astrovirus	Adenovirus non-41/41
Sapovirus	Parechovirus
Adenovirus type 40/41	COVID-19
Parasites	
Giardia lamblia	Microsporidium spp.
Cryptosporidium spp.	Blastocystis hominis*
Entamoeba histolytica	Isospora spp.
Dientamoeba fragilis	Cyclospora
Non-pathogenic parasites*	
Entamoeba gingivalis	Endolimax nana

Entamoeba hartmanni	Iodamoeba bütschlii
Entamoeba coli	Entamoeba dispar
Entamoeba polecki	Entamoeba moshkovskii
Other	
Parasitic worm eggs	Protozoan Cysts and Oocysts
Larvae	
**Serum screening**	
Hematology	
Complete Blood Count (CBC)	Gamma-glutamyl transferase (GGT)
Bilirubine	Alkaline phosphatase (AF)
C-reactive protein (CRP)	Kreatinine
Aspartate aminotransferase (ASAT)	Ureum
Alanine aminotransferase (ALAT)	Estimated Glomerular Filtration Rate (EGFR)
Bacteria	
Lues	
Viruses	
Hepatitis A	Cytomegalovirus (CMV)
Hepatitis B	Epstein-Barr Virus (EBV)
Hepatitis C	Human T-lymphotropic virus (HTLV)
Human immunodeficiency viruses (HIV)	
Parasites	
Strongyloides	

* Exclusion of donor only if microscopically "much" or "very much" blastocystis are seen
** Presence of only one non-pathogenic parasite is acceptable

CRE = Carbapenem-Resistant Enterobacteriaceae; ESBL = Extended spectrum beta-lactamase; MRGN = multidrug-resistant Gram-negative; MRSA = Methicillin-resistant Staphylococcus aureus; STEC = Shigatoxine-Producerende E. Coli; VRE = Vancomycin resistant enterococ; AF = Alkaline phosphatase; ALAT = Alanine aminotransferase; ASAT = Aspartate aminotransferase; CBC = complete blood count; CMV = cytomegalovirus; CRP = C-reactive protein; EGFR = Estimated Glomerular Filtration Rate; EBV = Epstein-Barr virus; GGT = Gamma-glutamyl transferase; HIV = Human immunodeficiency viruses; HTLV = Human T-lymphotropic virus

Eligibility criteria

Patients

Inclusion criteria

- Age 16-21 years
- Non-smokers
- Ability to give informed consent
- IBS diagnosis (Table 1)
- Refractory symptoms (Table 2)
- Average daily pain rate ≥30mm on the pain component scale of the IBS-SSS[34]

Exclusion criteria

Exclusion criteria are presented in Supplementary Table 1.

Donors

Inclusion criteria

- Age ≥16 years
- Non-smokers
- Ability to give informed consent
- BMI 18-25 kg/m2
- Regular morning stool pattern

Exclusion criteria

Exclusion criteria are presented in Supplementary Table 1.

Randomisation, blinding and treatment allocation

Confidential: For Review Only

Randomisation will be done by a computerised random-number generator in the Electronic Data Capture system Castor EDC in a 1:1 ratio to one of the following two treatment arms:

1. Allogeneic faecal infusions at t = 0 weeks and t = 6 weeks.
2. Autologous faecal infusions at t = 0 weeks and t = 6 weeks.

Randomly permuted blocks of size two and four will be used with no stratification. On the day of faecal transplantation, both patient and donors will deliver faeces produced that morning. Randomisation will be performed by one of the 'randomisation-assistants', who is designated to this task. To guarantee blinding, the randomisation-assistant will make sure the randomised treatment is not traceable to the donor or the patient. The blinded faeces will be brought to the Laboratory, where the preparation of the faeces will be done by one of the investigators. Detailed information about the preparation process is outlined under 'FMT procedure'. During the second FMT at 6 weeks, faeces will be processed according to the randomisation performed on the first transplantation day. The randomisation-assistant is the only person who will know which treatment the patient will be given and will have no role in further parts of the study. The randomisation list will be kept under secured access by Castor EDC. In case of an emergency, the study treatment can be unblinded after consultation of the principal investigator.

Intervention

FMT procedure

At baseline and at 6 weeks, patient and donor will collect a fresh morning stool sample in a small container and bring this to the AUMC for processing. Upon arrival of the patient in the hospital, a nasoduodenal tube will be positioned under direct imaging, with the Cortrak® electromagnetic sensing device.[35] After placement of the nasoduodenal tube, bowel lavage with 1.5–3.5 litres of macrogol electrolytes (Klean-Prep®) solution will be performed according to standard protocols to ensure complete bowel lavage. The amount of solution that is given depends on the rapidity by

Confidential: For Review Only

which the bowel is cleaned. Finally, a faecal suspension of 200cc will be infused in the duodenum of the patient through the nasoduodenal tube.

Preparation of faecal infusion product

On the day of infusion, a fresh faeces sample (100 – 200 g on average) of either the donor (allogeneic) or patient (autologous) will be used. In case a patient is not able to provide a fresh morning faeces sample, the first faecal production after the start of bowel-lavage with Klean-Prep ® is used as suitable faecal sample for further processing. Time of collection will be recorded. The faeces will be weighted and mixed with 200 – 400 ml Saline (0.9% NaCl) until fully homogenised. Next, the faeces solution is poured through a double gauze and debris of large size will be removed. This step will be repeated. Afterwards, the homogenised solution will be decanted through a metal funnel into a 200 cc sterile plastic bottle. All steps are performed under a fume-hood by one of the co-investigators. Within 6 hours after production by the donor, the faeces will be installed through the nasoduodenal tube in the patient.

Outcomes

All below mentioned outcome measures apply to patients.

Primary outcome

The primary objective of this RCT is to assess the feasibility of our study protocol. This will be assessed by evaluating the process of patient recruitment and screening, the patient drop-out rate and the incidence rates of adverse events. Table 5 delineates the feasibility outcome measures and measurement instruments.

Secondary outcomes

Secondary objectives include the proportion of patients with > 50% reduction of their abdominal pain intensity and pain frequency compared to baseline at t=12 weeks after the first FMT. This will be

https://mc.manuscriptcentral.com/bmjpo

Confidential: For Review Only

assessed with the pain component of the IBS-SSS.[34] Table 5 also describes all secondary outcome measures.

Confidential: For Review Only

Table 5. Trial outcome measures and instruments

	Outcome measures	Instrument
Feasibility outcomes	**Patient recruitment**	**Patient recruitment per month**<br>*patient/month recruited*
	**Patient screening**	**Patient eligibility**<br>*% of patients*
	**Patient drop-out**	**Patient drop-out rate after randomization**<br>*% of patients*, including patient acceptance to accomplish repetitive faecal microbiota transplantations (FMTs)
	**Serious adverse events related to FMT**	**Hospitalization or increase of > 100 points on pain component of IBS-SSS**<br>*% of patients*
	**Stool sample collection**	**Patients provide all necessary stool samples**<br>*% of the provided samples*, morning stool samples will be collected during all study visits
Efficacy outcomes	**> 50% reduction of abdominal pain intensity and pain frequency compared to baseline at 12 (T3), 24 (T5) and 48 (T6) weeks after first FMT**	**Pain component of Irritable Bowel Syndrome Severity Scoring System (IBS-SSS) score**[34]<br>With two questions, the severity and frequency of the abdominal pain on the last 10 days is measured. The IBS-SSS is the only symptom severity scale that has been responsive to treatment effects.[36] It has been recommended as a good instrument to obtain information on specific IBS related symptoms.[37]
	**Change in gut microbiota composition**	**MiSeq Illumina Sequencing**<br>Morning stool samples will be collected to profile the faecal microbiota composition by sequencing of the V4 region of the 16S ribosomal RNA (rRNA) gene
	**Change in gut mycobiome composition**	**ITS sequencing**<br>Morning stool sample will be collected to profile the faecal mycobiome composition by high-throughput rDNA sequencing of fungal internal transcribed spacer (ITS)-1 regions
	**Change in gut metabolome composition**	**Capillary electrophoresis time-of-flight mass spectrometry (CE-TOF-MS)**<br>Morning stool sample will be collected to profile the faecal metabolome composition by CE-TOF-MS
	**Number of adverse events**	**Patient CRF**
	**Number of rescue medication**	**Patient CRF**
	**Total IBS-SSS score**	**Irritable Bowel Syndrome Severity Scoring System (IBS-SSS) score**[34]<br>The IBS-SSS is the only symptom severity scale that has been responsive to treatment effects.[36] It has been recommended as a good instrument to obtain information on specific IBS related symptoms.[37]
	**Health related quality of life**	**Irritable Bowel Syndrome – Quality of Life (IBS-QOL) questionnaire**[38]<br>This questionnaire is a 34-item assessment of the degree to which the IBS interferes with patient quality of life and consists of eight domains: dysphoria, interference with activities, body image, health worry, food avoidance, social reactions, sexual health, and effect on relationships.[38]
	**Generic quality of life**	**Medical Outcomes Study 36-item Short Form Health Survey (SF-36)**<br>The SF-36 questionnaire consists of 36 questions regarding eight dimensions of health perception: limitations in physical functioning, role limitation due to physical health problems, bodily pain, general health perception, vitality, social functioning, role limitations due to emotional limitations, and mental health. A score between 0 (worst possible quality of life) and 100 (best possible quality of life) can be obtained. The reliability has been proven extensively for diverse patient groups and it is validated for the Dutch population.[39] The SF-36 is described as adequate for persons 14 years of age and older.[40]

**Depression and anxiety**	**Hospital Anxiety and Depression Scale (HADS)**<br>The HADS is divided into two 7-item scales, with answers on a four-point scale (0-3). Higher scores indicate a higher level of anxiety or depression (range 0-21). A scale score of ≥8 (cut-off score) indicates clinically significant anxiety or depression. The Dutch version of the HADS showed satisfactory validity and reliability.[41]
**Absence of school or work, healthcare resources and costs**	**Adapted version of the Dutch Health and Labor Questionnaire**[42]<br>School or work absenteeism and indirect healthcare utilization costs are measures by three items. Adolescents indicate whether they have been absent from school or work due to abdominal pain complaints, and if yes, the amount of hours per week.<br>For the indirect costs of healthcare utilization, adolescents indicate additional costs they had due to symptoms of abdominal pain over the past 4 weeks.
**Impact of treatment**	**Adapted version of the Patient Satisfaction and Preference Questionnaire**[43]<br>Impact of FMT treatment will be assessed using 5 questions, which are based on the Patient Satisfaction and Preference Questionnaire used in another RCT on FMT in patients with recurrent Clostridium Difficile infection.[43] The questions address thoughts on how unpleasant and how dirty participants find the idea of getting a faecal transplant.
**Adequate relief**	**One question:**<br>''Did you have adequate relief of IBS symptoms (abdominal discomfort/pain, bowel habits, and other symptoms like nausea and bloating) over the past week?'' (Yes/No)
**Plasma biomarkers**<br>– Intestinal fatty acid-binding protein (I-FABP)<br>– Smooth muscle protein of 22 kDa (SM-22)<br>– Citrulline	**Vena puncture**<br>EDTA vacuum tubes were used. All blood samples were centrifuged at 4000 rpm, at 41C for 15 minutes to obtain plasma. Plasma was immediately stored in aliquots at -80°C until analysis
**Safety parameters**<br>– C-reactive Protein (CRP)<br>– Liver function<br>– Renal function	**Vena puncture**<br>EDTA vacuum tubes were used. All blood samples were centrifuged at 4000 rpm, at 41C for 15 minutes to obtain plasma. Plasma was immediately stored in aliquots at -80°C until analysis
**Dietary intake**	**Dietary diary**<br>Dietary intake lists are filled out 7 days prior to each faecal sample collection.

CE-TOF-MS = capillary electrophoresis time-of-flight mass spectrometry; CRF = case report form; CRP = c-reactive protein; EDTA = ethylenediaminetetraacetic acid; FMT = faecal microbiota transplantation; HADS = Hospital Anxiety and Depression Scale; I-FABP = intestinal fatty acid-binding protein; IBS = irritable bowel syndrome; IBS-QOL = irritable bowel syndrome – quality of life; IBS-SSS = irritable bowel syndrome – severity scoring system; RCT = randomized controlled trial; rRNA = ribosomal RNA; SF-36 = Study 36-item Short Form Health Survey; SM-22 = smooth muscle protein of 22 kDa

nly

Participant timeline

Figure 1 displays the time schedule of enrolment, interventions, assessments and visits for participating patients.

Sample size calculation

Since this is a pilot study, a reliable sample size calculation is not feasible. In accordance with recruitment recommendations,[44,45] a minimum of 15 patients per treatment group will be included. In addition, based on accumulated evidence with 16S rRNA sequencing using MiSeq, Illumina Platform, a sample size of 20 individuals is normally enough to detect relevant differences in the microbiota. Hence, a total sample size of N=30 seems adequate. In order to reduce heterogeneity in faecal transplants, one donor will donate faeces to approximately 3 patients, which implicates that 5 donors are needed for 30 patients.

Statistical analysis

All data will be analysed according to the intention-to-treat principle. Feasibility outcome measures will be presented as proportions at each time point throughout the trial. To assess the efficacy outcomes group differences will be calculated by a mean difference with a 95% CI, using an independent t-test for continuous variables with a parametric distribution or Mann-Whitney U test for continuous variables with a non-parametric distribution. Group differences for categorical variables will be calculated using Fisher's exact statistics. In addition, data of continues variables will be analysed using mixed models to account for correlations of measurements within the same individual on several time points. Due to the small sample size, baseline values will not be incorporated in these analyses. Significance is set at $\alpha = 0.05$ in all analyses.

Microbiota composition of the faecal samples will be measured by 16S rRNA sequencing and specific genera/species are screened by qPCR. Alfa-and beta-diversity of faecal samples will be calculated. Cluster analysis and similarity of the microbiota profiles, expressed as Pearson correlation, will be

Confidential: For Review Only

assessed and compared between IBS patients and healthy donors, between treatment groups, and between responders and non-responders. In addition, short-chain fatty acids (SCFAs) composition of the faecal samples will be measured.

Monitoring

Data monitoring

In order to optimise safety of the study during inclusion, patient data will be disclosed to a data safety monitoring board (DSMB) when 50% of the intended sample size is attained and has reached 12 weeks follow up. The advice(s) of the DSMB will be notified upon receipt by the sponsor to the METC that approved the protocol. With this notification a statement will be included indicating whether the advice will be followed.

Harms

The risks associated with participation in this RCT can be considered moderate, because of the minimal invasive treatment. Nasoduodenal tube positioning through a Cortrak® electromagnetic sensing device carries a little risk of complications like aspiration, perforation or mal-positioning. If there is any doubt of malposition of the tube, a plain abdominal X-ray will be performed. To prevent complications, patients with swallowing disorders will not be included in this study.

Recent meta-analyses on clinical outcomes of FMT in general concluded that no serious adverse events were attributable to FMT.[30,46] Adverse events (AEs) were infrequent and mostly self-limiting (i.e. diarrhoea, abdominal distension, nausea and vomiting) and no differences existed in the number of AEs between donor FMT and control patients.[30,46] In our study, AEs will be monitored throughout the whole study. In order to make the risk for transmission of infectious diseases as small as possible rescreening of the faecal donors will be performed according to Table 6. In accordance to the legal requirements in the Netherlands (article 10, subsection 1, WMO), the investigator will

Confidential: For Review Only

inform the subjects and the reviewing accredited METC if harmful events occur. When there are indications that the disadvantage of participation may be significantly greater than was described in the research proposal, the study will be suspended pending a further positive decision by the accredited METC. The investigator will take care that all subjects are kept informed.

Confidential: For Review Only

Table 6. Time interval of donor rescreening*

	Rescreening interval			
	Pre-FMT	***4 weeks***	***8 weeks***	***26 weeks***
Short rescreening questionnaire	x			
Extensive rescreening questionnaire				x
**Faeces screening**				
Calprotectine				x
Bacteria				x
Antibiotic Resistant Bacteria COVID-19			x	
Viruses				x
Parasites				x
Non-pathogenic parasites**				x
Other				x
**Serum screening**				
Hematology				x
Bacteria				x
Viruses				x
Cytomegalovirus (CMV) *Epstein-Barr Virus (EBV)*		x*		
Parasites				x

* for specification of screening items see Table 4: specification of donor screening
** when a donor is seronegative for EBV IgG and/or CMV IgG

Ethics and dissemination

This study was approved by the Medical Ethics Research Committee of the AUMC in Amsterdam, the Netherlands. All important protocol amendments will be presented to the Medical Ethics Committee of the AUMC and will await approval before they are implemented.

Commencement of the trial

On November 23, 2017 the first study participant (in particular donor) was included in the trial. Until today, 58 potential donors were recruited of which 39 were included and started the screening procedure. Finally, a total of five donors were eligible to donate faeces. The first patient signed informed consent in August 2018. At time of writing 15 adolescents were recruited and a total of 19 faecal transplantations have been performed.

Review Only

DISCUSSION AND CONCLUSION

IBS is a chronic and disabling condition, which can pose great impact on daily life of patients, reflected in decreased quality of life,[4,5] high work or school absence,[6,7] a higher risk to develop depressive and anxiety disorders.[7,8] and substantial healthcare costs.[9,10] Effective management strategies for adolescents and adults in the form of antidepressants, peppermint oil, cognitive behavioural therapy, hypnotherapy, probiotics and low FODMAP diet exist. However, a subgroup of IBS patients remains symptomatic. New effective treatment options for this subgroup are warranted and might be targeted on the altered microbiome in IBS patients.[24]

Up to now, six RCTs have been performed to assess the effect of FMT in IBS in adults. Two trials assessed the effect of FMT administered by capsules, two evaluated the effect of FMT delivered by colonoscopy, one via gastroscope, and one by nasojejunal tube.[31] It appears that the efficacy of FMT is associated with the methodology of FMT and placebo, as donor faeces administered by colonoscopy, gastroscopy or nasojejunal tube demonstrated a clinically significant improvement in global IBS symptoms in comparison with autologous FMT via the same route, whereas stool capsules did not demonstrate any beneficial effect compared to placebo capsules.[31]

The present pilot study assesses the feasibility of FMT in adolescents with refractory IBS according to the Rome IV criteria. Furthermore, the efficacy of FMT on abdominal pain symptoms in these patients is explored. By designing this specific treatment protocol, a unique opportunity is created to investigate potential beneficial effects of restoring the gut microbiota composition on abdominal pain complaints. Data of this study will help determine optimal study conditions and inform the choice of endpoints for future, larger size, double-blind RCTs on FMT in adolescents with IBS. Furthermore, this study will define preliminary efficacy results of the use of FMT in these patients. In addition, this study will enable us to analyse in detail which microbiota components might predict a positive response to FMT.

Confidential: For Review Only

Our study has several strengths. First, the FMT will be administered via a nasoduodenal tube and it will be performed twice, since it has been demonstrated that this might enhance the effect of the FMT.[47] Another strength is the one-year follow-up, which allows us to assess the long-term effect of FMT.

A limitation of our study is the small sample size, which allows us to only encounter major effects of the FMT treatment. Furthermore, we decided to include IBS patients regardless of subtype, leading to a heterogeneous patient population which may affect the efficacy results. Moreover, it is unclear what the effect of bowel lavage is on the efficacy of FMT and on microbiome composition. Studies with and without bowel preparations before FMT demonstrate great efficacy.[47,48] In addition, it has been shown that bowel preparation can disrupt the colonic ecosystem were the overall microbiome composition recovers to baseline within 14 days after bowel cleansing.[49] Our efficacy outcome measure is assessed at 12 weeks after the first FMT (and 6 weeks after the second FMT), which minimises the effect that the bowel cleansing can have on the microbiome composition.

In conclusion, the results of this trial will provide preliminary evidence for the use of FMT in adolescents with refractory IBS. The results will inform future larger, double-blind, placebo-controlled trials on the right sample size, on the feasibility of this study design, on efficacy outcome measures and on the potential of the microbiome to be a therapeutic target in IBS.

Confidential: For Review Only

Contributorship Statement

MAB is the principal investigator, designed the study, wrote the protocol, supervised the trial, and supervised drafting of the manuscript. JZ participated in the design of the study, wrote the protocol, coordinated part of the trial, and was responsible for data collection, analysis and drafting the manuscript. CMAB coordinates the trial, and is responsible for data collection, analysis and drafting the manuscript. AV and MN contributed to the design of the trial, critically revised the protocol, and supervised drafting of the manuscript.

Funding

This research received no specific grant from any funding agency in the public, commercial or not-for-profit sectors.

Competing interests

M. Nieuwdorp is in the scientific advisory board of Caelus Health and Kaleido BioSciences; however, none of these are directly related to the current manuscript.

The other have no competing interests relevant to this article to disclose.

Acknowledgements

Dr. I.J.N. Koppen and dr. D.R. Hoekman provided substantial conceptual contributions. Prof. dr. A.H. Zwinderman provided contributions to statistical considerations for the trial.

Confidential: For Review Only

REFERENCES

1 Sperber AD, Dumitrascu D, Fukudo S, *et al.* The global prevalence of IBS in adults remains elusive due to the heterogeneity of studies: a Rome Foundation working team literature review. *Gut* 2017;**66**:1075–82. doi:10.1136/gutjnl-2015-311240

2 Korterink JJ, Diederen K, Benninga MA, *et al.* Epidemiology of Pediatric Functional Abdominal Pain Disorders: A Meta-Analysis. *PLoS One* 2015;**10**:e0126982. doi:10.1371/journal.pone.0126982

3 Sagawa T, Okamura S, Kakizaki S, *et al.* Functional gastrointestinal disorders in adolescents and quality of school life. *J Gastroenterol Hepatol* 2013;**28**:285–90. doi:10.1111/j.1440-1746.2012.07257.x

4 Gralnek IM, Hays RD, Kilbourne A, *et al.* The impact of irritable bowel syndrome on health-related quality of life. *Gastroenterology* 2000;**119**:654–60.

5 Youssef NN. Quality of Life for Children With Functional Abdominal Pain: A Comparison Study of Patients' and Parents' Perceptions. *Pediatrics* 2006;**117**:54–9. doi:10.1542/peds.2005-0114

6 Canavan C, West J, Card T. Review article: the economic impact of the irritable bowel syndrome. *Aliment Pharmacol Ther* 2014;**40**:1023–34. doi:10.1111/apt.12938

7 Youssef NN, Atienza K, Langseder AL, *et al.* Chronic Abdominal Pain and Depressive Symptoms: Analysis of the National Longitudinal Study of Adolescent Health. *Clin Gastroenterol Hepatol* 2008;**6**:329–32. doi:10.1016/j.cgh.2007.12.019

8 Fond G, Loundou A, Hamdani N, *et al.* Anxiety and depression comorbidities in irritable bowel syndrome (IBS): a systematic review and meta-analysis. *Eur Arch Psychiatry Clin Neurosci* 2014;**264**:651–60. doi:10.1007/s00406-014-0502-z

9 Brandt LJ, Chey WD, Foxx-Orenstein AE, *et al.* An Evidence-Based Position Statement on the Management of Irritable Bowel Syndrome. *Am J Gastroenterol* 2008;**104**:S1–35. doi:10.1038/ajg.2008.122

10 Hoekman DR, Rutten JMTM, Vlieger AM, *et al.* Annual Costs of Care for Pediatric Irritable Bowel Syndrome, Functional Abdominal Pain, and Functional Abdominal Pain Syndrome. *J Pediatr* 2015;**167**:1103-1108.e2. doi:10.1016/j.jpeds.2015.07.058

11 Hyams JS, Di Lorenzo C, Saps M, *et al.* Childhood Functional Gastrointestinal Disorders: Child/Adolescent. *Gastroenterology* 2016;**150**:1456-1468.e2. doi:10.1053/j.gastro.2016.02.015

12 Drossman DA. The functional gastrointestinal disorders and the Rome III process. *Gastroenterology* 2006;**130**:1377–90. doi:10.1053/j.gastro.2006.03.008

13 Rutten JMTM, Korterink JJ, Venmans LMAJ, *et al.* [Guideline on functional abdominal pain in children]. *Ned Tijdschr Geneeskd* 2017;**161**:D781.

14 Ford AC, Lacy BE, Harris LA, *et al.* Effect of Antidepressants and Psychological Therapies in Irritable Bowel Syndrome. *Am J Gastroenterol* 2019;**114**:21–39. doi:10.1038/s41395-018-0222-5

15 Black CJ, Yuan Y, Selinger CP, *et al.* Efficacy of soluble fibre, antispasmodic drugs, and gut–brain neuromodulators in irritable bowel syndrome: a systematic review and network meta-

https://mc.manuscriptcentral.com/bmjpo

Confidential: For Review Only

analysis. *Lancet Gastroenterol Hepatol* Published Online First: December 2019. doi:10.1016/S2468-1253(19)30324-3

16 Martin AE, Newlove-Delgado T V, Abbott RA, *et al.* Pharmacological interventions for recurrent abdominal pain in childhood. In: Martin AE, ed. *Cochrane Database of Systematic Reviews*. Chichester, UK: : John Wiley & Sons, Ltd 2017. CD010973. doi:10.1002/14651858.CD010973.pub2

17 Ford AC, Harris LA, Lacy BE, *et al.* Systematic review with meta-analysis: the efficacy of prebiotics, probiotics, synbiotics and antibiotics in irritable bowel syndrome. *Aliment Pharmacol Ther* 2018;**48**:1044–60. doi:10.1111/apt.15001

18 Newlove-Delgado T V, Martin AE, Abbott RA, *et al.* Dietary interventions for recurrent abdominal pain in childhood. *Cochrane database Syst Rev* 2017;**3**:CD010972. doi:10.1002/14651858.CD010972.pub2

19 Dionne J, Ford AC, Yuan Y, *et al.* A Systematic Review and Meta-Analysis Evaluating the Efficacy of a Gluten-Free Diet and a Low FODMAPS Diet in Treating Symptoms of Irritable Bowel Syndrome. *Am J Gastroenterol* 2018;**113**:1290–300. doi:10.1038/s41395-018-0195-4

20 Abbott RA, Martin AE, Newlove-Delgado T V, *et al.* Psychosocial interventions for recurrent abdominal pain in childhood. *Cochrane database Syst Rev* 2017;**1**:CD010971. doi:10.1002/14651858.CD010971.pub2

21 Vlieger AM, Rutten JMTM, Govers AMAP, *et al.* Long-term follow-up of gut-directed hypnotherapy vs. standard care in children with functional abdominal pain or irritable bowel syndrome. *Am J Gastroenterol* 2012;**107**:627–31. doi:10.1038/ajg.2011.487

22 Saulnier DM, Riehle K, Mistretta T-A, *et al.* Gastrointestinal microbiome signatures of pediatric patients with irritable bowel syndrome. *Gastroenterology* 2011;**141**:1782–91. doi:10.1053/j.gastro.2011.06.072

23 Tap J, Derrien M, Törnblom H, *et al.* Identification of an Intestinal Microbiota Signature Associated With Severity of Irritable Bowel Syndrome. *Gastroenterology* 2017;**152**:111-123.e8. doi:10.1053/j.gastro.2016.09.049

24 Pittayanon R, Lau JT, Yuan Y, *et al.* Gut Microbiota in Patients With Irritable Bowel Syndrome—A Systematic Review. *Gastroenterology* 2019;**157**:97–108. doi:10.1053/j.gastro.2019.03.049

25 Hyland NP, Quigley EMM, Brint E. Microbiota-host interactions in irritable bowel syndrome: epithelial barrier, immune regulation and brain-gut interactions. *World J Gastroenterol* 2014;**20**:8859–66. doi:10.3748/wjg.v20.i27.8859

26 Öhman L, Törnblom H, Simrén M. Crosstalk at the mucosal border: importance of the gut microenvironment in IBS. *Nat Rev Gastroenterol Hepatol* 2015;**12**:36–49. doi:10.1038/nrgastro.2014.200

27 van Nood E, Vrieze A, Nieuwdorp M, *et al.* Duodenal infusion of donor feces for recurrent Clostridium difficile. *N Engl J Med* 2013;**368**:407–15. doi:10.1056/NEJMoa1205037

28 Costello SP, Hughes PA, Waters O, *et al.* Effect of Fecal Microbiota Transplantation on 8-Week Remission in Patients With Ulcerative Colitis. *JAMA* 2019;**321**:156. doi:10.1001/jama.2018.20046

Confidential: For Review Only

29 Kootte RS, Levin E, Salojärvi J, *et al.* Improvement of Insulin Sensitivity after Lean Donor Feces in Metabolic Syndrome Is Driven by Baseline Intestinal Microbiota Composition. *Cell Metab* 2017;**26**:611-619.e6. doi:10.1016/j.cmet.2017.09.008

30 Xu D, Chen VL, Steiner CA, *et al.* Efficacy of Fecal Microbiota Transplantation in Irritable Bowel Syndrome. *Am J Gastroenterol* 2019;**114**:1043–50. doi:10.14309/ajg.0000000000000198

31 Ianiro G, Eusebi LH, Black CJ, *et al.* Systematic review with meta-analysis: efficacy of faecal microbiota transplantation for the treatment of irritable bowel syndrome. *Aliment Pharmacol Ther* 2019;**50**:240–8. doi:10.1111/apt.15330

32 El-Salhy M, Hatlebakk JG, Gilja OH, *et al.* Efficacy of faecal microbiota transplantation for patients with irritable bowel syndrome in a randomised, double-blind, placebo-controlled study. *Gut* 2019;:gutjnl-2019-319630. doi:10.1136/gutjnl-2019-319630

33 Terveer EM, van Beurden YH, Goorhuis A, *et al.* How to: Establish and run a stool bank. *Clin Microbiol Infect* 2017;**23**:924–30. doi:10.1016/j.cmi.2017.05.015

34 Francis CY, Morris J, Whorwell PJ. The irritable bowel severity scoring system: a simple method of monitoring irritable bowel syndrome and its progress. *Aliment Pharmacol Ther* 1997;**11**:395–402.

35 Rao MM, Kallam R, Flindall I, *et al.* Use of Cortrak--an electromagnetic sensing device in placement of enteral feeding tubes. *Proc Nutr Soc* 2008;**67**:E109. doi:10.1017/S0029665108007416

36 Irvine EJ, Tack J, Crowell MD, *et al.* Design of Treatment Trials for Functional Gastrointestinal Disorders. *Gastroenterology* 2016;**150**:1469-1480.e1. doi:10.1053/j.gastro.2016.02.010

37 Bijkerk CJ, de Wit NJ, Muris JWM, *et al.* Outcome measures in irritable bowel syndrome: comparison of psychometric and methodological characteristics. *Am J Gastroenterol* 2003;**98**:122–7. doi:10.1111/j.1572-0241.2003.07158.x

38 Patrick DL, Drossman DA, Frederick IO, *et al.* Quality of life in persons with irritable bowel syndrome: development and validation of a new measure. *Dig Dis Sci* 1998;**43**:400–11.

39 Aaronson NK, Muller M, Cohen PD, *et al.* Translation, validation, and norming of the Dutch language version of the SF-36 Health Survey in community and chronic disease populations. *J Clin Epidemiol* 1998;**51**:1055–68.

40 Ware JE. SF-36 health survey update. *Spine (Phila Pa 1976)* 2000;**25**:3130–9.

41 Spinhoven P, Ormel J, Sloekers PP, *et al.* A validation study of the Hospital Anxiety and Depression Scale (HADS) in different groups of Dutch subjects. *Psychol Med* 1997;**27**:363–70.

42 Van Roijen L, Essink-bot M-L, Koopmanschap MA, *et al.* Labor and Health Status in Economic Evaluation of Health Care: The Health and Labor Questionnaire. *Int J Technol Assess Health Care* 1996;**12**:405–15. doi:10.1017/S0266462300009764

43 Kao D, Roach B, Silva M, *et al.* Effect of Oral Capsule– vs Colonoscopy-Delivered Fecal Microbiota Transplantation on Recurrent *Clostridium difficile* Infection. *JAMA* 2017;**318**:1985. doi:10.1001/jama.2017.17077

44 Sim J, Lewis M. The size of a pilot study for a clinical trial should be calculated in relation to considerations of precision and efficiency. *J Clin Epidemiol* 2012;**65**:301–8.

Confidential: For Review Only

doi:10.1016/j.jclinepi.2011.07.011

45 Lancaster GA, Dodd S, Williamson PR. Design and analysis of pilot studies: recommendations for good practice. *J Eval Clin Pract* 2004;**10**:307–12. doi:10.1111/j.2002.384.doc.x

46 Lai CY, Sung J, Cheng F, *et al.* Systematic review with meta-analysis: review of donor features, procedures and outcomes in 168 clinical studies of faecal microbiota transplantation. *Aliment Pharmacol Ther* 2019;**49**:354–63. doi:10.1111/apt.15116

47 Ianiro G, Maida M, Burisch J, *et al.* Efficacy of different faecal microbiota transplantation protocols for Clostridium difficile infection: A systematic review and meta-analysis. *United Eur Gastroenterol J* 2018;**6**:1232–44. doi:10.1177/2050640618780762

48 Postigo R, Kim JH. Colonoscopic versus nasogastric fecal transplantation for the treatment of Clostridium difficile infection: a review and pooled analysis. *Infection* 2012;**40**:643–8. doi:10.1007/s15010-012-0307-9

49 Nagata N, Tohya M, Fukuda S, *et al.* Effects of bowel preparation on the human gut microbiome and metabolome. *Sci Rep* 2019;**9**:4042. doi:10.1038/s41598-019-40182-9

Confidential: For Review Only

Address correspondence

Judith Zeevenhooven, Emma Children's Hospital, Amsterdam UMC, University of Amsterdam, Paediatric Gastroenterology, Room C2-312, PO Box 22700, 1100 DD Amsterdam, The Netherlands.
Telephone: +3120-5662906. Email: j.zeevenhooven@amsterdamumc.nl

Confidential: For Review Only

Figure legends

Figure 1. Trial design. After adolescents sign the informed consent form (T-2), patients complete the baseline pain diary, the IBS-SSS and deliver stool samples and blood samples for eligibility screening. At T0, adolescents are randomised in the allogeneic or autologous FMT group.

Confidential: For Review Only

	Enrolment	Allocation	During treatment		Follow-up, *Number of weeks after first FMT*			
**TIMEPOINT**	**T -2**	**T0**	**T1**	**T2**	**T3**	**T4**	**T5**	**T6**
	**-2 weeks**	**Baseline**	**3 weeks**	**6 weeks**	**12 weeks**	**16 weeks**	**24 weeks**	**48 weeks**
	**Screening patient**	**First FMT**		**Second FMT**				
**ENROLMENT:**								
AMC visit	X	X		X	X		X	X
Phone assessment			X			X		
Eligibility screen	X							
Informed consent	X							
Allocation		X						
**INTERVENTIONS:**								
Allogeneic FMT		⟷		⟷				
Autologous FMT		⟷		⟷				
**ASSESSMENTS:**								
Pain diary card	X							
IBS-SSS	X	X	X	X	X	X	X	X
Morning stool sample	X	X		X	X		X	X
Blood samples (20 ml)	X	X		X	X			
Adverse events			X	X	X	X	X	X
Questionnaires:<br>- Quality of life<br>- Depression/anxiety<br>- School/work absenteeism<br>- Impact of treatment<br>- Adequate relief		X		X	X		X	X
Dietary booklet	X	X		X	X		X	X

Figure 1. Trial design

After adolescents sign the informed consent form (T-2), patients complete the baseline pain diary, the IBS-SSS and deliver stool samples and blood samples for eligibility screening. At T0, adolescents are randomised in the allogeneic or autologous FMT group.

FMT = fecal microbiota transplantation; IBS-SSS = Irritable Bowel Syndrome Severity Scoring System

Confidential: For Review Only

**Supplementary Table 1. Exclusion criteria patients and donors**
**Patients**
Use of systemic antibiotics in preceding 6 weeks
Use of probiotic treatment in preceding 6 weeks
Use of concomitant medication, including proton pomp inhibitors (PPI) and vasopressine medication. Pain medication in the form of Paracetamol or NSAIDs is allowed.
Current use of drugs which influence gastrointestinal motility (erythromycin, azithromycin, butyl scopolamine, domperidone, peppermint oil capsules, iberogast)
Current treatment by another health care professional for abdominal symptoms
Current treatment by psychologist or shrink for known anxiety or depression disorder
Known swallowing disorder
Known diagnosis of inflammatory bowel disease (i.e. Crohn's disease or ulcerative colitis)
Known concomitant organic gastrointestinal disease
Known diagnosis of an autoimmune disease (e.g. hypo- or hyperthyroidism, celiac disease, rheumatoid arthritis)
Condition leading to profound immunosuppression (HIV, infectious diseases leading to immunosuppression, bone marrow malignancies)
Known diagnosis of cystic fibrosis
Known diagnosis of porphyria
Known pregnancy or current lactation
Use of systematic chemotherapy
Life expectancy < 12 months
Current Intensive Care Unit-stay
XTC, amphetamine or cocaine abuse
Known intra-abdominal fistula
Signs of ileus, diminished passage
Allergy to macrogol or substituents, e.g. peanuts, shellfish
History of surgery:<br>o *Hemicolectomy (defined as: surgery resulting in a resection of > 0.5 of the colon)*<br>o *Presence of a pouch due to surgery*<br>o *Presence of stoma*
Insufficient knowledge of the Dutch language
**Donors**
Abnormal bowel motions, abdominal complaints or symptoms indicative of irritable bowel syndrome
An extensive travel behaviour
Higher risk of colonization with multidrug- resistant organisms including:<br>o *Health care workers*<br>o *Persons who have recently been hospitalized or discharged from long term care facilities*<br>o *Persons who regularly attend outpatient medical or surgical clinics*<br>o *Persons who have recently engaged in medical tourism*
Unsafe sex practice (assessed with standardized questionnaire)
Use of any medication including PPI
Antibiotic treatment in the past 12 weeks
A positive history/clinical evidence for inflammatory bowel disease (i.e. Crohn's disease or ulcerative colitis)
A positive history/clinical evidence for other gastrointestinal diseases, including chronic diarrhoea or chronic constipation

Patients receiving immunosuppressive medications or a positive history/clinical evidence for autoimmune disease including:<br>o *Type 1 diabetes*<br>o *Hashimoto hypothyroidism*<br>o *Graves hyperthyroidism*<br>o *Rheumatoid arthritis*<br>o *Celiac disease*
History of or present known malignant disease and/or patients who are receiving systemic anti-neoplastic agents
Known psychiatric disease (i.e. depression, schizophrenia, autism, Asperger's syndrome)
Known chronic neurological/neurodegenerative disease (e.g. Parkinson's disease, multiple sclerosis)
Positive blood tests for the presence of: HIV, HTLV, lues, Strongyloides
Active hepatitis A, B-, C- or E-virus infection or known exposure within recent 12 months
Acute infection with cytomegalovirus (CMV) or Epstein-Barr virus (EBV)
Chronic pain syndromes (e.g. fibromyalgia)
Major relevant allergies (e.g. food allergy, multiple allergies)
Recent (gastrointestinal) infection within last 6 months
Tattoo or body piercing placement within last 6 months
Alcohol abuse (>3 units/day)
Known risk of Creutzfeldt Jacob's disease
History of current use of IV drugs
History of treatment with growth factors

For Review Only
